# Transpupillary in vivo two-photon imaging reveals enhanced surveillance of retinal microglia in diabetic mice

**DOI:** 10.1073/pnas.2426241122

**Published:** 2025-10-08

**Authors:** Noriyuki Sotani, Sentaro Kusuhara, Ryuto Nishisho, Hiroto Kuno, Hidenori Shima, Koichiro Haruwaka, Yuka Mori, Maya Kishi, Tomoyuki Furuyashiki, Kenta Kobayashi, Hiroaki Wake, Toru Takumi, Makoto Nakamura, Yoshihisa Tachibana

**Affiliations:** ^a^Department of Physiology and Cell Biology, Kobe University Graduate School of Medicine, Kobe 650-0017, Japan; ^b^Division of Ophthalmology, Department of Surgery, Kobe University Graduate School of Medicine, Kobe 650-0017, Japan; ^c^Center for Neuroimmunology and Glial Biology, Institute of Molecular Medicine, University of Texas Health Science Center, Houston, TX 77030; ^d^Division of Pharmacology, Kobe University Graduate School of Medicine, Kobe 650-0017, Japan; ^e^Section of Viral Vector Development, National Institute for Physiological Sciences, Okazaki 444-8585, Japan; ^f^Department of Anatomy and Molecular Cell Biology, Nagoya University Graduate School of Medicine, Nagoya 466-8550, Japan

**Keywords:** two-photon microscopy, retina, microglia, diabetes mellitus, liraglutide

## Abstract

The retina, located outside the cranium, serves as an ideal structure for investigating information processing within the central nervous system due to its well-organized neurovascular unit comprising diverse cell types, including neurons, glial cells (such as microglia, Müller glia, and astrocytes), pericytes, and vascular endothelial cells. Disrupted retinal homeostasis contributes to various ocular diseases such as diabetic retinopathy, age-related macular degeneration, glaucoma, and retinitis pigmentosa. However, noninvasive in vivo imaging methods to study the pathogenesis of these disorders remain limited. Here, we developed a two-photon microscopy technique for real-time, transpupillary in vivo visualization of the retinal neurovascular unit in mice. Our approach integrates systematic head fixation, a custom-made polymethyl methacrylate contact lens, and a glycerin immersion objective lens with an extended working distance and a higher numerical aperture, specifically designed for two-photon microscopy. This method enabled us to visualize dynamic microglial process activity around retinal blood vessels. Our results revealed that retinal microglia exhibit enhanced surveillance under diabetic conditions, which is undetectable by static confocal microscopy. Furthermore, we demonstrated that liraglutide, a glucagon-like peptide-1 receptor agonist commonly used for the treatment of diabetes and obesity, reversed the enhanced microglial behaviors in the diabetic retina. Our simple yet effective approach mitigates the need for advanced optical systems like adaptive optics, providing an effective tool for real-time imaging of the retina. This method offers a valuable resource for visual neuroscience research and holds great potential for clinical applications, particularly in the early diagnosis, intervention, and monitoring of retinal diseases.

## Significance

Numerous studies have developed imaging techniques for visualizing diverse cell types in the retina. However, these techniques often face challenges such as low resolution and the need for technically demanding setups. To overcome these limitations, we developed a transpupillary in vivo two-photon retinal imaging method that combines systemic head fixation, a custom-made contact lens, and a glycerin immersion objective lens with a long working distance. Using this method, we captured clear images of the retinal neurovascular unit and visualized enhanced microglial surveillance in diabetic retinas. Furthermore, we demonstrated that liraglutide, a diabetes medication, modulated microglial motility toward patterns observed in nondiabetic controls. This accessible technique bridges basic research and clinical applications, advancing the early detection and treatment of ocular disorders.

The retina, located outside the cranium, is a unique component of the central nervous system (CNS). It is composed of a wide variety of cells, including neurons, glial cells (such as microglia, Müller glia, and astrocytes), retinal pigment epithelial cells, pericytes, and vascular endothelial cells (1, 2). This cellular diversity makes the retina an excellent model for investigating information processing within the CNS.

Disrupted homeostatic regulation of the retinal environment can result in various ocular diseases such as diabetic retinopathy, age-related macular degeneration, glaucoma, and retinitis pigmentosa (3–6). In vivo real-time retinal imaging using two-photon microscopy can be a powerful tool for visualizing retinal components in both animal models and humans, providing insights into the pathogenesis of such diseases. Although several retinal imaging studies in mice using two-photon microscopy have recently been reported (7–13), technical challenges remain. These include limited resolution due to the small numerical aperture of the objective lenses, as well as the technical demands of adaptive optics. To overcome these limitations, we developed a straightforward method for visualizing retinal components (neurons, glial cells, and blood vessels) in living mice. Our approach employs adeno-associated viruses (AAVs) and transgenic mice, along with a custom-made polymethyl methacrylate (PMMA) contact lens combined with a glycerin immersion objective lens that has a long working distance. This method enables high-resolution imaging of the retina without the need for adaptive optics.

To evaluate the capability of our imaging method, we focused on the behavior and morphological changes of microglia in a mouse model of diabetes. Microglia are resident immune cells in the CNS, derived from yolk sac progenitors. These cells play crucial roles in responding to infection, injury, and inflammation in the brain, and constantly surveying the environment to maintain homeostasis (14, 15). Microglia are also known to dramatically alter their processes depending on physiological or pathological (inflammatory) conditions (16). Diabetic retinopathy, a vision-threatening diabetic complication, has been historically attributed to vascular damage in the retina, leading to subsequent neuronal death. However, emerging evidence suggests that microglial and astrocytic activation due to hyperglycemia may precede retinal capillary dropout and breakdown of the blood–retinal barrier (BRB) (17–19).

The neurovascular unit (NVU), composed of neurons, glial cells, pericytes, and vascular endothelial cells, plays a critical role in maintaining CNS homeostasis, including the formation and maintenance of BRB (20). Dysfunction in the NVU may contribute to the breakdown of the BRB and retinal neurodegeneration. Notably, recent studies suggest that retinal neurodegeneration can occur prior to morphological vascular changes in diabetic eyes, highlighting the importance of investigating NVU abnormalities (21–23). Among the NVU components, microglia are key immune cells that actively survey the retinal environment and respond rapidly to hyperglycemic states. Therefore, this study aims to investigate microglial responses to hyperglycemia by comparing their process dynamics in normal and diabetic mellitus (DM) mouse retinas. To generate the mouse model of diabetes, we administered streptozotocin (STZ), which selectively destroys pancreatic insulin-producing β-cells, mimicking the hyperglycemic conditions of diabetes (24).

Here, we present a noninvasive, real-time transpupillary in vivo imaging method for visualizing NVU in the mouse retina. This method employs a simple head fixation, a custom-made contact lens, and an objective lens with an extended working distance and a higher numerical aperture, optimized for two-photon microscopy. Using this approach, we revealed enhanced microglial surveillance in STZ-treated diabetic mice. Importantly, we demonstrated that liraglutide, a diabetes medication, modulated microglial motility to levels similar to those observed in nondiabetic controls.

## Results

### Development of Stabilized Transpupillary Retinal Imaging Platform for Two-Photon Microscopy.

**Fig. 1.**
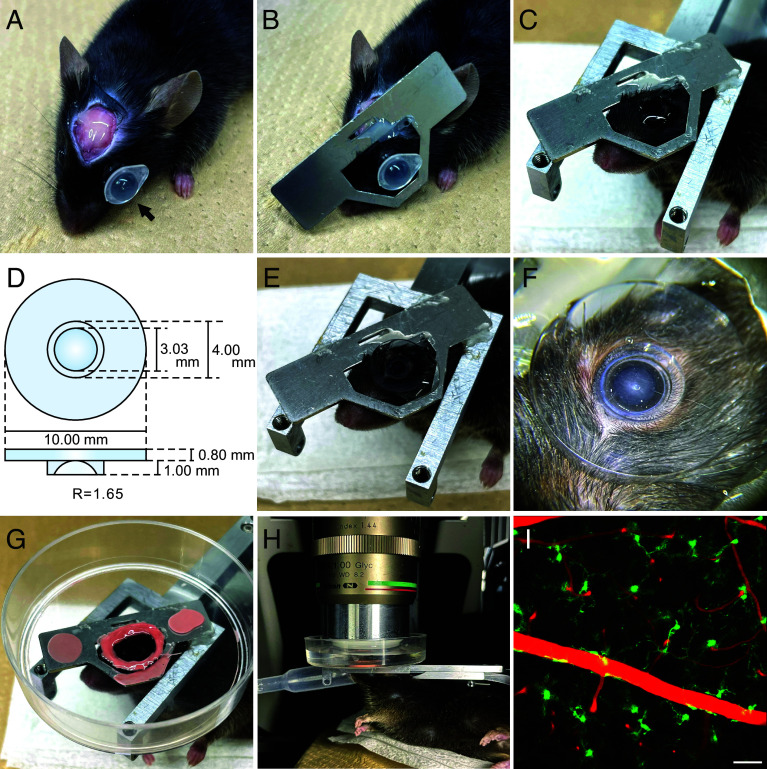
Experimental setup for transpupillary in vivo mouse retinal imaging under two-photon microscopy. (*A*) Application of dental cement to the exposed skull and placement of cataract-preventing cap (indicated by a black arrow) on the eyeball. The ophthalmic gel was applied beneath the cap. (*B*) Securing a custom-made metal plate to the skull. (*C*) Mounting the mouse on the fixation apparatus. (*D*) Design and specifications of the custom-made contact lens. (*E*) Placement of the contact lens on the mouse’s eyeball. (*F*) Magnified view of (*E*). (*G*) Attachment of a glycerin reservoir (adapted from a cell culture dish) to the fixation apparatus. (*H*) Side view of in vivo retinal imaging of an isoflurane-anesthetized mouse under two-photon microscopy, using a glycerin immersion objective lens. (*I*) Representative image of in vivo retinal observation showing microglia (green fluorescence labeled with GFP) and blood vessels (red fluorescence labeled with Evans blue) in a Cx3cr1^GFP/+^ mouse. (Scale bar, 50 µm.)

Fig. 1 illustrates the detailed procedure for stabilized retinal observation through the pupil of a living Cx3cr1^GFP/+^ mouse, in which microglia express GFP (25). The scalp of a ketamine-anesthetized mouse was removed after applying local anesthesia with epinephrine-containing lidocaine to minimize bleeding. Strong resin cement (*SI Appendix,* Table S1) was applied to the exposed skull (Fig. 1*A*). To prevent cataract formation, we placed a sufficient amount of hydroxyethyl cellulose gel between the cornea and a plastic cap adapted from a standard PCR tube (indicated by an arrow in Fig. 1*A*). After a metal plate was attached to the resin cement on the skull (Fig. 1*B*), the mouse was secured in a fixation apparatus (Fig. 1*C*). During this process, the head plate was carefully aligned to ensure that the eyeball remained in its natural position, facing vertically, which was critical for achieving consistent and undistorted retinal imaging.

We designed a plano-concave PMMA contact lens with an ultrasmooth polished surface, explicitly optimized for two-photon microscopy in the mouse retina (Fig. 1*D*; see *Materials and Methods* for detailed lens specifications). After positioning the contact lens on the cornea (Fig. 1 *E* and *F*), a cell culture dish with a hollowed-out center, designed to fit around the contact lens, was secured to the fixation apparatus (Fig. 1*G*). The gap between the culture dish and contact lens was sealed using dental impression silicone. The contact lens was gently placed on the corneal surface, ensuring that only its own weight provided the necessary pressure for adhesion. This approach, combined with careful fixation using the silicone holder, minimized mechanical stress and preserved the cornea’s natural shape, essential for distortion-free imaging conditions.

To optimize the visualization of retinal elements under two-photon microscopy, we evaluated several commercially available objective lenses and selected a glycerin immersion objective lens (Fig. 1*H*; ×20, NA 1.0) due to its unique ability to achieve a long working distance (8.2 mm) solely through its internal optical design. This mitigates the need for external attachments or adaptive optics, which can introduce additional optical surfaces and alignment complexities, potentially degrading image quality. During this setup, the head fixation apparatus was carefully adjusted to ensure that the contact lens surface remained parallel to the objective lens plane, thereby maintaining the alignment of the optical axis and ensuring uniform imaging quality across the field of view (FOV).

These carefully designed and implemented steps enabled visualization of microglial morphology (green fluorescence) in an isoflurane-anesthetized Cx3cr1^GFP/+^ mouse, with retinal blood vessels (red fluorescence) visualized by intraperitoneal injection of Evans blue dye (Fig. 1*I*). Movie S1 demonstrates the dynamic movements of microglial processes around blood vessels over a 30-min recording (scanning speed: one image every 4 s). Furthermore, by combining viral injections with intraperitoneal dye administration or using fluorescence-expressing transgenic mice, we achieved simultaneous in vivo imaging of blood vessels with vascular endothelial cells, retinal ganglion cells with microglia, Müller glia with blood vessels, and astrocytes with microglia (*SI Appendix,* Fig. S1 *A*–*D*).

Compared with previously reported methods (9, 12, 13), our approach integrates meticulous head fixation, a custom-designed contact lens, and a glycerin immersion objective lens with an extended working distance. This configuration achieves surface precision without the need for additional adaptive optics systems. These advancements facilitate consistent imaging across the retinal FOV while effectively minimizing spherical aberrations, which is crucial for high-resolution imaging under two-photon microscopy. To evaluate the performance of our two-photon imaging system, we measured the lateral and axial resolutions by calculating the full width at half maximum (FWHM) of the line and point spread functions, using microglial processes as a reference structure. The averaged resolutions obtained from three independent measurements were 1.38 μm laterally and 19.80 μm axially (*SI Appendix*, Fig. S1 *E*–*H*). These values are comparable to the median values reported for two-photon imaging setup with system adaptive optics (correction of aberrations in the microscope’s optics alone) or full adaptive optics (correction of both the microscope and ocular aberrations) (9).

### Static Morphological Analysis of Retinal Microglia in Diabetic Mice.

**Fig. 2.**
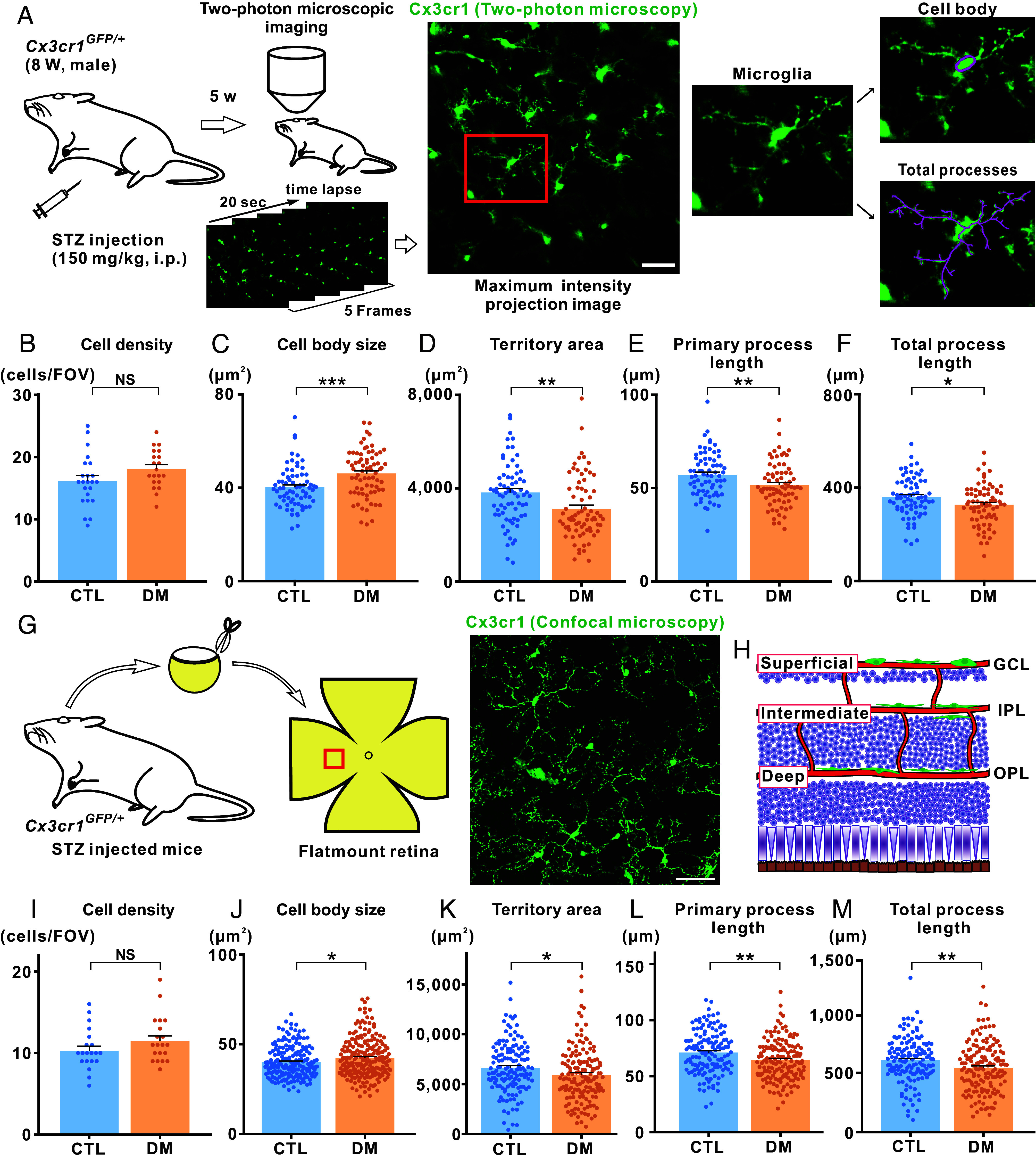
Static analysis of morphological changes in retinal microglia of diabetic mice. (*A*) Experimental timeline of STZ-treated DM model and two-photon microscopy imaging of retinal microglia. A representative in vivo two-photon image of retinal microglia from a diabetic Cx3cr1^GFP/+^ mouse is shown. (Scale bar, 50 µm.) The maximum intensity projection image was generated from five continuous images (4-s scanning each, totaling 20 s of scanning). The area indicated by a red rectangle in the maximum intensity projection image is enlarged to highlight the cell body (*Top* panel, far right) and all processes (*Bottom* panel, total processes) of a single microglial cell. (*B*–*F*) Quantitative data on microglial morphology in control (CTL) and DM mice based on two-photon microscopy images. (*B*) Density of microglial cells per FOV. A total of 23 and 20 FOVs from nine mice were analyzed in the CTL and DM groups, respectively. (*C*–*F*) Cell body size (*C*), territory area (*D*), primary process length (*E*), and total process length (*F*) of individual microglial cells. For these parameters, 72 microglial cells from nine mice were analyzed for each group. (*G*) Preparation of a flat-mounted retina from a diabetic Cx3cr1^GFP/+^ mouse. A representative confocal microscopy image of retinal microglia (captured from a red rectangle in the flat-mounted retina) is shown. (Scale bar, 50 µm.) (*H*) Schematic drawing depicting the location of microglia (green) in relation to blood vessels (red) at three different depths: superficial, intermediate, and deep. GCL, ganglion cell layer. IPL, inner plexiform layer. OPL, outer plexiform layer. (*I*–*M*) Quantitative data on microglial morphology in CTL and DM mice based on confocal microscopy images. (*I*) Cell density per FOV. A total of 20 FOVs from five mice were analyzed for each group. (*J*–*M*) Cell body size (*J*), territory area (*K*), primary process length (*L*), and total process length (*M*) of individual microglial cells. For these parameters, 195 and 216 microglial cells (*J*), 142 and 153 cells (*K*–*M*) from five mice were analyzed in the CTL and DM groups, respectively. Two-photon and confocal images were sampled from the superficial and intermediate layers in the retina. NS, not significant; *P < 0.05; **P < 0.01; ***P < 0.001 (Welch’s *t* test; Mann–Whitney U test was applied only for cell density in *I*). Data are presented as mean ± SEM.

To examine the static morphology of retinal microglia in diabetic mice, we employed our stabilized transpupillary retinal imaging technique in both control and STZ-treated diabetic mice. It is important to note that separate cohorts of mice were used for each experimental condition in this and subsequent analyses. A single intraperitoneal injection of STZ (150 mg/kg) typically elevated blood glucose levels to over 300 mg/dL within 1 wk (26). In this study, we used STZ-treated mice that maintained blood glucose levels above 400 mg/dL 5 wk after the injection (*SI Appendix,* Table S2 and Fig. S2) and performed two-photon microscopy to visualize retinal microglia (Fig. 2*A*). The retinal image was acquired via a 4-s scan at specific depths, and maximum intensity projection images were generated from five consecutive scans (totaling 20 s of scanning). Using these maximum intensity projection images, we compared the morphology of microglial cell bodies and processes between control and STZ-treated diabetic mice.

Retinal microglia are distributed horizontally along the blood vessels within three distinct retinal layers: the ganglion cell layer (GCL, superficial), the inner plexiform layer (IPL, intermediate), and the outer plexiform layer (OPL, deep) (Fig. 2*H*) (27). Prior to conducting quantitative analyses of retinal microglia captured by two-photon microscopy, we verified whether the GFP-positive cells in Cx3cr1^GFP/+^ mice were yolk-sac-derived microglia or infiltrating macrophages. To this end, we performed immunohistochemistry using antibodies against P2Y12R (a purinergic receptor specific to resident microglia) and CD169 (a marker for monocytes or infiltrating macrophages) (28). Confocal microscopy revealed that the majority of GFP-positive cells were P2Y12R-positive, indicating resident microglia, whereas only a small subset exhibited CD169 immunoreactivity (*SI Appendix*, Fig. S3 *A* and *B*). Notably, the proportion of CD169-positive cells among all GFP-positive cells did not significantly differ between control and diabetic mice, suggesting that diabetic conditions did not increase macrophage infiltration (*SI Appendix*, Fig. S3*C*). Interestingly, the proportion of CD169-positive in the intermediate and deep layers was smaller than in the superficial layer. Morphologically, CD169-positive cells displayed an ameboid shape with shorter primary processes (*SI Appendix*, Fig. S3*D*) and a highly polarized appearance, distinguishing them from the ramified morphological feature of CD169-negative homeostatic microglia. Based on these findings, we selectively analyzed GFP-positive cells displaying a ramified microglial morphology, presumably resident microglia, in subsequent quantitative analyses of two-photon and confocal microscopy data.

Group analysis of GFP-positive cells, sampled from the superficial and intermediate retinal layers, revealed no significant differences in the cell density of microglia in two-photon microscopy images (1,024 × 1,024 pixels) between control and diabetic mice (Fig. 2*B*). However, the cell body size of microglia was significantly larger in diabetic mice compared with controls (Fig. 2*C*). Next, we evaluated the morphology of microglial processes using two-photon microscopy. Diabetic mice exhibited significant reductions in the territory area (Fig. 2*D*), primary process length (Fig. 2*E*), and total process length (Fig. 2*F*) of microglial cells compared with controls. These group-level results were derived from layer-specific data, in which similar trends were observed, although statistical significance was not always reached for individual parameters (*SI Appendix*, Table S3 and Fig. S4 *A* and *B*). Collectively, static morphological analyses of retinal microglia demonstrated an increase in cell body size and a reduction in processes under diabetic conditions when assessed in vivo.

To further validate these findings, we examined the morphology of retinal microglia using confocal microscopy images of flat-mounted retinas (Fig. 2*G*; see also *Materials and Methods*). Confocal microscopy images were sampled from the superficial, intermediate, and deep layers of retinal vasculature (Fig. 2*H*). However, for group-level comparisons with two-photon microscopy data, only the images from the superficial and intermediate layers were used. The analysis revealed consistent results with two-photon microscopy across all assessed morphological parameters (Fig. 2 *I*–*M*). Further layer-specific analyses, including the data from the deep layer, demonstrated similar trends, with differences between control and diabetic mice becoming more pronounced from superficial to deep layers (*SI Appendix*, Fig. S4 *C*–*E*).

Furthermore, Cx3cr1^GFP/+^ mice possess only one copy of the Cx3cr1 gene, potentially affecting microglial function. To address this concern, we conducted additional immunohistochemical analyses using flat-mounted retinal tissues. Specifically, we compared microglial morphology between two control groups (i.e., Cx3cr1^+/+^ wild-type versus Cx3cr1^GFP/+^ mice) by performing immunohistochemistry using antibodies against IBA1 (a marker for both microglia and macrophages) and CD68 (a lysosome marker expressed in activated microglia or phagocytic macrophages) (29). Our analysis revealed no significant differences in morphological features (*SI Appendix*, Fig. S5*A*) and phagocytic activity (*SI Appendix*, Fig. S5*C*) between the two groups. These results suggest that the majority of microglia in Cx3cr1^GFP/+^ mice maintain a homeostatic, noninflammatory state. In contrast, diabetic mice exhibited markedly increased CD68 immunoreactivity compared with controls, reflecting increased phagocytic activity (*SI Appendix*, Fig. S5 *B* and *D*).

### Dynamic Properties of Retinal Microglial Processes in Diabetic Mice.

**Fig. 3.**
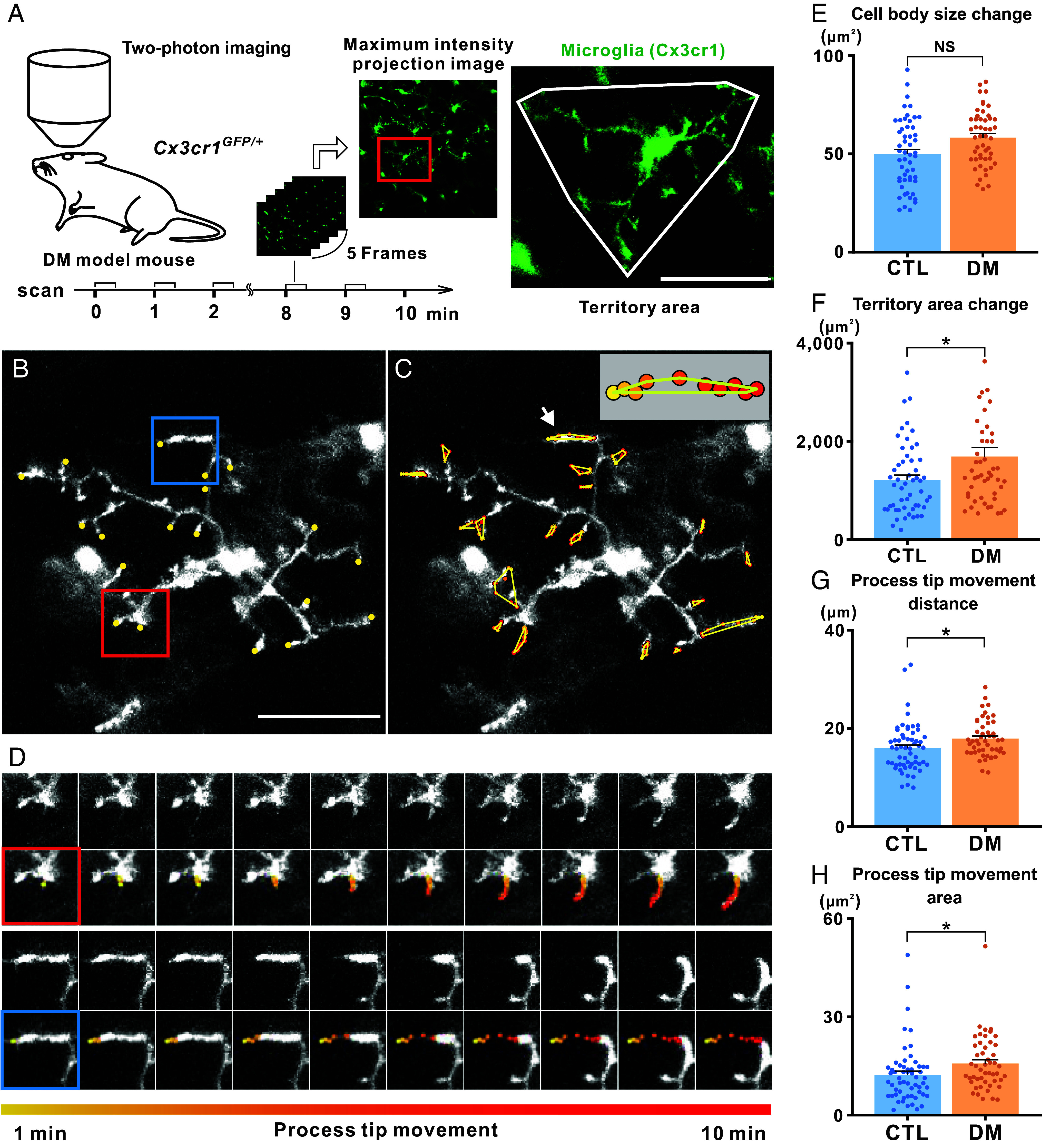
Dynamic properties of retinal microglia in diabetic mice observed via two-photon microscopy. (*A*) Experimental timeline of sequential (10-min) two-photon microscopy imaging of retinal microglia. The maximum intensity projection image was generated from five continuous images, in the same manner as described in Fig. 2*A*. Acquisition of the maximum intensity projection image was repeated for 10 min. The area indicated by a red rectangle in the maximum intensity projection image is enlarged to show the territory area occupied by all processes of a single microglial cell (indicated by a white triangle in the *Right* panel). (*B*–*D*) Representative two-photon microscopy images showing dynamic movements of multiple processes of a single microglial cell from an STZ-treated Cx3cr1^GFP/+^ DM. (*B*) Individual tips of microglial processes at a specific time point are marked with yellow dots. (Scale bar, 50 µm.) (*C*) 10-min trajectories of individual tips of microglial processes. Each colored dot represents the location of a microglial process tip at a different time point, showing its movement over the observation period. *Inset*: the 10-min surveillance territory of the tip of a microglial process (indicated by a white arrow), surrounded by a yellow polygon. (*D*) Magnified view of the 10-min trajectories of two representative processes of a single microglial cell, indicated by the red and blue rectangles in (*B*). The top two rows (red rectangle) show the elongation of a microglial process in the lower-left direction, while the bottom two rows (blue rectangle) show the retraction of another microglial process in the right direction. The first and third rows show the original images of the two processes, while the second and fourth rows highlight the tips of the processes, marked by colored dots showing the transition from yellow (1st time point) to red (10th time point). (*E*–*H*) Quantitative data showing dynamic changes in microglial morphology in control (CTL) and DM mice based on two-photon microscopy images. A total of 58 and 49 microglial cells, sampled from the superficial and intermediate retinal layers from seven mice, were analyzed in the CTL and DM groups, respectively. The data in (*E*–*H*) were obtained from the summation of changes over 10 min in cell body size (*E*), territory area occupied by all processes of a single microglial cell (*F*), movement distance of process tips (*G*), and surveillance territory area of process tips (*H*). The datasets shown in (*E*–*H*) were obtained from the same cohorts analyzed in Fig. 2 *B*–*F*. NS, not significant; *P < 0.05 (Welch’s *t* test). Data are presented as mean ± SEM.

In our static morphological analysis using two-photon imaging in control and diabetic mice, we observed significant differences in morphological parameters of microglial processes (such as the territory area, primary process length, and total process length) at a single time point. Furthermore, microglia are known to be highly dynamic cells, capable of extending and retracting their processes to continuously survey the brain environment (Movie S1). Therefore, we hypothesized that retinal microglial processes might exhibit more dynamic behaviors under pathological conditions such as diabetes. To test this hypothesis, we conducted sequential two-photon imaging of microglia processes over a continuous 10-min period (Fig. 3*A*). Sequential observation of individual retinal microglia in diabetic mice highlighted dynamic process movements (Fig. 3*B*, initial positions of individual processes; Fig. 3*C*, sequential observations over 10 min; see also Movie S2). Fig. 3*D* provides magnified views of two representative processes from the microglia shown in Fig. 3*B*. One process extended toward the lower-left direction (top two rows, indicated by a red rectangle; the initial point in yellow, and the end point in red). Another process retracted toward the right direction (bottom two rows, indicated by a blue rectangle).

To evaluate the dynamic properties of microglia over the 10-min period, we calculated several parameters, including the change in cell body size (Fig. 3*E*), the change in the territory area occupied by all processes of a single microglial cell (Fig. 3*F*), the movement distance of process tips (Fig. 3*G*), and the change in surveillance territory area of process tips (Fig. 3*H*, as defined in the inset of Fig. 3*C*). Although the change in cell body size did not differ significantly between groups (Fig. 3*E*), diabetic mice exhibited a significantly larger degree of change in the territory area occupied by individual microglial cells (Fig. 3*F*), as well as increased movement distance of process tips (Fig. 3*G*) and an expanded surveillance territory area of process tips (Fig. 3*H*) compared with controls. Next, we calculated the extension and retraction speeds of microglial processes in control and diabetic mice. The extension speed in diabetic mice was slightly higher than that in controls, but the difference did not reach statistical significance (*SI Appendix*, Table S3 and Fig. S6). In contrast, the retraction speed was significantly higher in diabetic mice compared with controls. Notably, there was no significant difference between the extension and retraction speeds of microglial processes within diabetic mice. These findings suggest that microglia in diabetic mice exhibit enhanced dynamic surveillance activity, including faster retraction speed (see also Movie S2).

To further validate the capability of our two-photon imaging system for detecting microglial motility, we examined microglial dynamics in a well-established model of systemic inflammation induced by lipopolysaccharide (LPS) injection (see also *SI Appendix*, Fig. S5 *B* and *D*). The increases in the movement distance and surveillance territory area of microglial process tips observed in LPS-injected mice were comparable to those observed in diabetic mice (*SI Appendix*, Fig. S7).

### Antidiabetic Drug Reverses Enhanced Movement Properties of Microglia in Diabetic Mice.

**Fig. 4.**
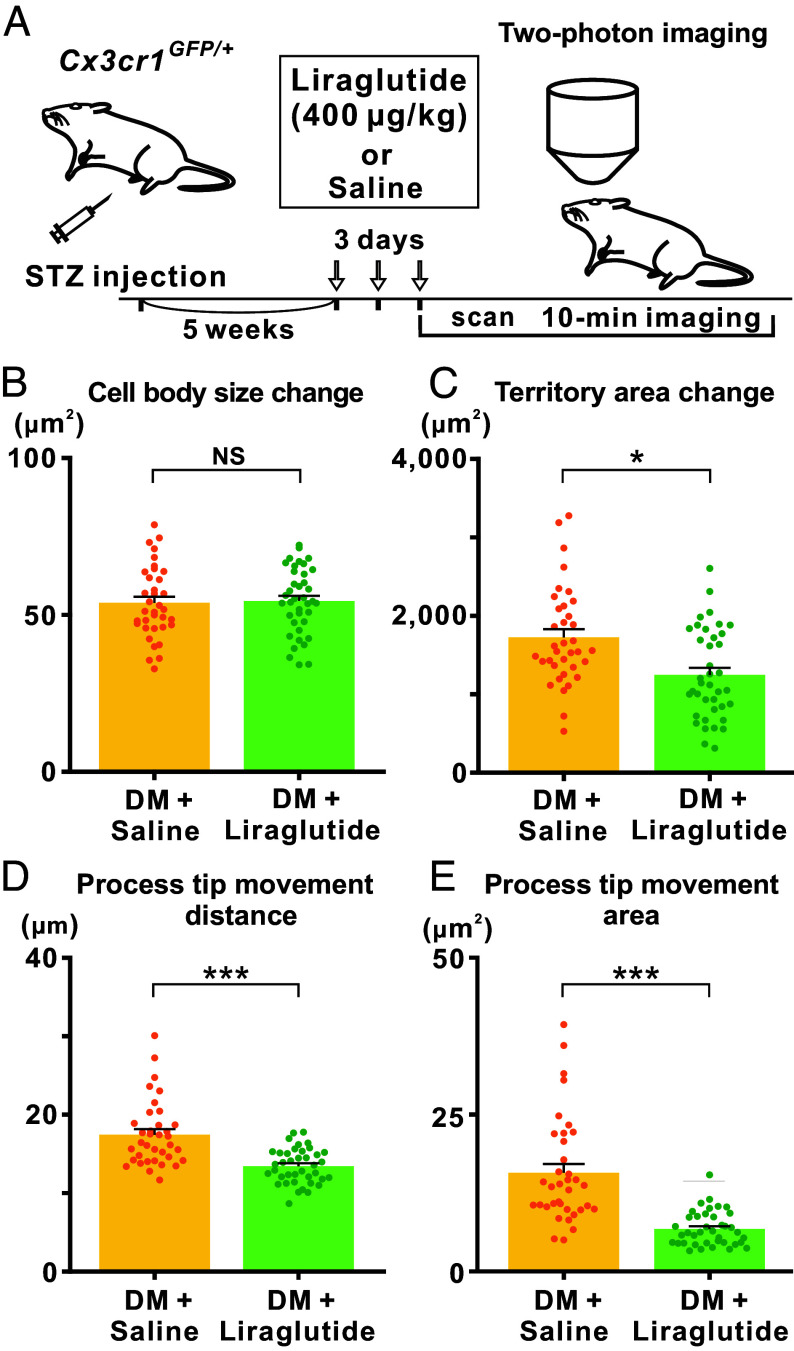
Liraglutide reverses enhanced movement properties of microglia in diabetic mice, as observed via two-photon microscopy. (*A*) Experimental timeline of liraglutide or saline administration in STZ-treated DM, followed by two-photon microscopy imaging of retinal microglia. (*B*–*E*) Quantitative data showing the dynamic movement properties of microglial morphology in saline-injected (DM + Saline) and liraglutide-injected diabetic mice (DM + Liraglutide), based on two-photon microscopy images. A total of 36 and 39 microglial cells, sampled from the superficial and intermediate retinal layers from five mice, were analyzed in the DM + Saline and DM + Liraglutide groups, respectively. The data in (*B*–*E*) represent the summation of changes over a 10-min period in cell body size (*B*), territory area (*C*), movement distance of process tips (*D*), and surveillance territory area of process tips (*E*). The datasets for the DM + Saline and DM + Liraglutide groups shown in (*B*–*E*) were obtained from different cohorts used in Fig. 3. NS, not significant; *P < 0.05; ***P < 0.001 (Welch’s *t* test). Data are presented as mean ± SEM.

We next investigated whether liraglutide, a glucagon-like peptide-1 (GLP-1) receptor agonist commonly used for the treatment of diabetes and obesity, could normalize the enhanced microglial surveillance observed in diabetic mice (Fig. 4*A*). We compared the dynamic properties of microglial morphology between two groups: 1) STZ-treated diabetic mice receiving subcutaneous saline injections, and 2) STZ-treated diabetic mice receiving subcutaneous liraglutide injections. Although 3 consecutive days of liraglutide administration did not lower blood glucose levels (*SI Appendix,* Table S2 and Fig. S2), we hypothesized that the drug could exert direct or indirect effects on retinal microglia (*Discussion*). While liraglutide treatment did not alter the change in microglial cell body size (Fig. 4*B*), it significantly suppressed the diabetes-induced increase in microglial movement. Specifically, liraglutide administration reduced the enhanced changes in the territory area of individual microglia (Fig. 4*C*), the movement distance of process tips (Fig. 4*D*), and the surveillance territory area of process tips (Fig. 4*E*).

To validate the effects of liraglutide on retinal microglia, we performed confocal microscopic analysis to compare microglial morphology between saline-injected and liraglutide-treated diabetic mice. Our analysis revealed that morphological alterations induced by diabetes were not only reversed but also restored to levels beyond those observed in nondiabetic controls (*SI Appendix*, Table S3 and Fig. S8). Moreover, microglial phagocytic activity, as assessed by CD68 immunoreactivity, was similarly restored to the control level following liraglutide administration (*SI Appendix*, Fig. S5*D*).

To further explore whether liraglutide affects microglial surveillance activity independently of hyperglycemia, we also administered liraglutide to nondiabetic mice. Interestingly, we found that liraglutide treatment reduced microglial motility in nondiabetic mice, with the suppressive effect being slightly stronger in diabetic mice (*SI Appendix*, Fig. S9 *A*–*D* compared with Fig. 4; see also *SI Appendix,* Table S3). Confocal microscopy analysis further revealed significant morphological changes only in primary process length, although the direction of these changes was opposite to that seen under diabetic conditions (*SI Appendix*, Fig. S9 *E*–*I*). Taken together, these findings indicate that liraglutide suppresses retinal microglial surveillance activity under both nondiabetic and diabetic conditions, consistent with a glucose-independent mechanism. The greater magnitude of suppression observed in the diabetic state further suggests that diabetes-associated factors may enhance this effect.

Finally, we investigated the possible involvement of pericytes, mural cells that wrap around endothelial cells within the retinal NVU, in regulating microglial surveillance activity. Previous studies have demonstrated that pericyte depletion disrupts microglial homeostasis in the developing brain (30). In addition, diabetes is known to impair pericyte function in the brain, and this dysfunction can be reversed by GLP-1 receptor agonists (31). Based on these findings, we hypothesized that liraglutide might restore pericyte function in diabetic retinas, thereby contributing to the normalization of microglial surveillance activity. To test this hypothesis, we immunostained retinal pericytes using an anti-NG2 antibody in nondiabetic controls, untreated diabetic mice, and liraglutide-treated diabetic mice (*SI Appendix*, Fig. S10). However, quantitative analysis revealed no significant differences in the density of NG2-positive cells (presumably pericytes) around blood vessels among the three groups. These findings suggest that the suppressive effect of liraglutide on microglial surveillance activity is unlikely to be mediated through pericyte function.

## Discussion

Dysregulation of retinal microglia has been reported in patients and animal models of various ocular diseases (18, 32–35). To understand the physiological and pathological roles of microglia within the CNS, it is essential to examine the dynamic properties of microglia under in vivo conditions. In this study, our high-resolution two-photon microscopy imaging revealed that retinal microglia exhibit altered surveillance activity in diabetic states, and liraglutide treatment reversed these diabetes-induced alterations in microglial dynamics.

### Two-Photon Microscopy Observation of Retinal Components.

Two-photon microscopy is a powerful tool for investigating intact brain tissues in living animals. This technique is particularly well-established for visualizing neuronal layers in the cerebral cortex, typically located 300 to 500 µm below the dural surface (36, 37). In contrast, the mouse retina is positioned approximately 3 mm from the corneal surface (38). Despite this greater depth, two-photon microscopy is advantageous for retinal imaging due to the transparency of the cornea, lens, and vitreous body to infrared light. Several studies have already applied two-photon microscopy to image the mouse retina, focusing on visualizing retinal neurons and blood vessels under in vivo conditions (7–13).

Nevertheless, technical challenges remain, including limited resolution arising from the small numerical aperture of the objective lens for deep tissue imaging. Recent advances in two-photon microscopy with adaptive optics have achieved subcellular-resolution imaging of microglial dynamics in the retina. For example, Qin et al. (9) and Zhang et al. (13) demonstrated active microglial motility in the retina using systems equipped with deformable mirrors and wavefront sensors to correct optical aberrations. While highly effective, these adaptive optics systems require specialized equipment and sophisticated technical expertise, limiting their broader applicability.

To overcome these limitations, we developed a more straightforward technique for visualizing retinal components that would be accessible to researchers and clinicians who may not have access to adaptive optics setups. Our system achieved a stable, depth-resolved two-photon imaging using a long-working-distance, high-NA glycerin immersion objective combined with a custom plano-concave PMMA contact lens, without the need for adaptive optics. This setup enables longitudinal imaging with sufficient spatial resolution (*SI Appendix*, Fig. S1) and excitation powers within safety limits, making it broadly applicable and cost-effective. Importantly, our approach is not mutually exclusive with adaptive optics; future integration of our system with adaptive optics could further enhance image quality.

In addition to two-photon microscopy, high-resolution optical coherence tomography (OCT) and confocal scanning laser ophthalmoscopy (SLO) (39–42) have been widely used to image retinal microglia, particularly in Cx3cr1^GFP/+^ mice (40, 42). While SLO offers the advantage of noninvasive, longitudinal imaging over large retinal areas, it suffers from limited depth resolution and lacks the ability to resolve fine microglial processes or capture detailed dynamic behaviors. Moreover, confocal SLO is associated with significant thermal energy deposition, which can potentially induce photothermal damage during prolonged imaging sessions. Our two-photon imaging approach addresses these limitations by providing precise and detailed images of microglial shape and activity, especially under disease conditions such as diabetes.

### Functional and Morphological Changes in Retinal Microglia in Diabetic States.

Hyperglycemia-induced activation of retinal microglia has been reported to promote the production of both neuroprotective mediators and proinflammatory cytokines in diabetic states (18, 19, 32), similar to observations in brain microglia of diabetic mice (43). An imbalance between these opposing factors leads to neuronal degeneration. Specifically, the production of neurotoxic inflammatory factors, such as tumor necrosis factor-α (TNF-α), interleukin-6 (IL-6), glutamate, oxidative stress, and nitrous oxide, results in the dysfunction of neurons as well as damage to pericytes and endothelial cells (33, 44). Since the retina functions as a specialized NVU, understanding hyperglycemia’s impact on microglia and other cellular components of the NVU is crucial.

Microglia have emerged as key regulators of the NVU, capable of modulating both vascular structure and barrier function. For example, capillary-associated microglia have been shown to stabilize capillary diameter through purinergic P2Y12 receptor signaling (45), and their ablation impairs the rapid closure of blood–brain barrier (BBB) breaches, indicating that microglial processes actively participate in sealing leaky vessels and preserving barrier function (46). In addition to microglia, the retinal NVU contains two types of macroglia: Müller glia and astrocytes. Under physiological conditions, Müller glia are essential for the modulation of neurotransmitters (uptake and recycling), regulation of blood flow, and maintenance of the BRB, whereas astrocytes provide nutritional and regulatory support (18, 47). However, under diabetic conditions, chronic activation of microglia can drive perivascular microglia to phagocytose astrocytic end-feet and release cytokines, leading to BBB breakdown (16). This highlights the dual effects of microglial interaction with vascular and macroglial components, being beneficial under healthy conditions but potentially destructive during disease states (45, 46, 48, 49). These dual mechanisms are particularly evident in diabetic retinopathy, where microglial activation and perivascular clustering exacerbate vascular leakage and inflammation (50).

Our analysis also revealed that the density of pericytes wrapping around endothelial cells, assessed by NG2 immunostaining, was not significantly different among control, diabetic, and liraglutide-treated mice (*SI Appendix*, Fig. S10). These findings suggest that while the density of pericytes remains stable under diabetic conditions, potential microglia-mediated functional shifts in pericyte behavior cannot be ruled out. Future studies should explore these microglial–pericyte interactions to clarify their contributions to the pathogenesis of diabetic retinopathy.

A previous study has demonstrated that retinal microglia in Alloxan-induced diabetic mice display larger cell body sizes and shorter processes than those in control mice (51). This study also demonstrated that microglial cell density across different layers of the retina remained unchanged between control and diabetic mice. Our confocal and two-photon imaging data corroborate these findings; while microglial cell density remained constant, diabetic mice exhibited enlarged cell bodies and shortened microglial processes compared with controls (Fig. 2).

Interestingly, another study using STZ-treated diabetic rats did not detect significant changes in microglial morphology (24). This discrepancy may be due to differences in species, as well as in the STZ dosage protocol. In our study, a higher STZ dose was used in mice, which may induce more pronounced retinal inflammation and microglial activation. Our two-photon microscopy data further support these diabetes-associated alterations in retinal microglia (Fig. 2).

More importantly, two-photon imaging allowed us to highlight enhanced microglial surveillance dynamics in diabetic mice. Diabetic mice exhibited significantly greater changes in the territory area of a single microglial cell (Fig. 3*F*), increased movement distance of process tips (Fig. 3*G*), and increased change in surveillance territory area of process tips (Fig. 3*H*) compared with controls. These findings indicate that hyperglycemia not only induces morphological changes in retinal microglia but also markedly enhances their dynamic surveillance activity, revealing an important aspect of pathophysiology in diabetic retinopathy.

### Potential Therapeutic Effects of Antidiabetic Drug on Activated Retinal Microglia.

We have demonstrated that liraglutide, an antidiabetic drug, can normalize the enhanced surveillance activity of retinal microglia under diabetic conditions. Liraglutide, a GLP-1 receptor analog, is known to promote insulin secretion from pancreatic β-cells in response to hyperglycemia and is widely used to control blood glucose levels in patients with type 2 diabetes. However, in STZ-treated diabetic mice, pancreatic β-cells are destroyed mainly, and liraglutide does not typically reduce blood glucose levels in this model. Consistent with this, liraglutide treatment did not alter blood glucose levels in our STZ-treated diabetic mice (*SI Appendix,* Table S2 and Fig. S2). This finding rules out the possibility that the observed effects of liraglutide on retinal microglia are attributable to improvements in hyperglycemia. While variability in the extent of pancreatic β-cell destruction among STZ-treated mice could occasionally result in reduced blood glucose levels in some individuals treated with liraglutide, our data showed no such changes (*SI Appendix,* Fig. S2), effectively excluding this possibility. In addition, our analysis demonstrated that the liraglutide altered the surveillance activity of retinal microglia even in nondiabetic mice (*SI Appendix,* Fig. S9). These results provide strong evidence that liraglutide modulates retinal microglial activity independently of blood glucose levels.

GLP-1 receptors are widely expressed in multiple organs, including the human retina, brain, heart, lung, and pancreas (52–54). Beyond its well-known benefits for glycemic control, multiple studies have demonstrated that liraglutide can directly target microglia and modulate their neuroinflammatory phenotype (55). Although the expression of GLP-1 receptors in retinal microglia and macroglia (i.e., Müller glia and astrocytes) remains debated (52), liraglutide may alleviate the proinflammatory effects of retinal microglia through direct or indirect mechanisms (54). For example, GLP-1 receptor activation has been shown to suppress the secretion of proinflammatory cytokines such as TNF-α, IL-6, and IL-1β in cultured microglia (56). Furthermore, liraglutide has been reported to reduce the number of activated microglia and levels of TNF-α and IL-6 in the hippocampus of a palmitate-induced inflammatory mouse model (57). While previous studies have focused on liraglutide’s ability to modulate microglial activation markers and cytokine release, little is known about its impact on the dynamic behavior of microglial processes in vivo.

Our two-photon microscopy data demonstrate that liraglutide can normalize the heightened surveillance activity of retinal microglia in diabetic mice. Importantly, the elevated phagocytic activity observed in diabetic retinas, evidenced by increased CD68 immunoreactivity, which is also elevated in human diabetic retinopathy (58), was significantly reduced following liraglutide treatment. However, it remains unresolved whether the enhanced microglial motility reflects pathological or protective effects on the retinal NVU across the course of disease progression. To address this issue, future longitudinal two-photon imaging studies will be required to directly visualize the release of proinflammatory cytokines and neuroprotective mediators within the retinal NVU. Such studies could provide deeper insights into liraglutide’s potential as a therapeutic agent for diabetic retinopathy.

## Materials and Methods

### Animals.

All procedures were approved by the Institutional Animal Care and Use Committees of Kobe University. Female wild-type C57BL/6J mice were crossed with male Cx3cr1^GFP/GFP^ mice (Jackson Laboratory, Stock No: 005582) to generate Cx3cr1^GFP/+^ offspring for all experiments to visualize microglia. Although a larger number of mice were initially prepared, five Cx3cr1^GFP/GFP^ and 57 Cx3cr1^GFP/+^ mice (all male, aged 8 to 15 wk) were included in the final analyses based on imaging quality. All of the animals in this study were given free access to food and water and housed under a 12-h light/dark cycle.

### Experimental Model of Diabetes and Systemic Inflammation.

Eight- to ten-wk-old Cx3cr1^GFP/+^ mice received a single intraperitoneal injection of STZ (150 mg/kg; Nacalai Tesque, Kyoto, Japan) dissolved in 50 mM citrate buffer (pH 4.5) to induce hyperglycemia (24). Blood glucose levels were measured using a portable glucometer (Medisafe Fit, Terumo, Tokyo, Japan) 1 and 5 wk after the injection. Mice with blood glucose levels exceeding 400 mg/dL in both tests were considered diabetic mice, and included in the study. Control mice received an intraperitoneal injection of the vehicle (citrate buffer). To establish an additional mouse model of systemic inflammation, a single intraperitoneal injection of lipopolysaccharide (LPS; 9 mg/kg; Sigma-Aldrich, St. Louis, MO) was administered 5 h prior to the head fixation (see below).

### Head Fixation and Contact Lens Mounting.

Diabetic and control mice were anesthetized via intraperitoneal injection of ketamine (150 mg/kg, i.p.; Ketalar, Daiichi Sankyo, Tokyo, Japan) and xylazine (22.5 mg/kg, i.p.; Seractar, Elanco Japan, Tokyo, Japan). Ophthalmic hydroxyethyl cellulose gel (Scopisol, Senju Pharmaceutical, Osaka, Japan) was applied to the cornea to prevent cataract formation, which can deteriorate the image quality of microglial morphology. This was followed by the placement of a moisture-preserving plastic cap adapted from a standard PCR tube (Fig. 1*A*). The frontal head region was shaved, and after subcutaneous injection of lidocaine hydrochloride containing epinephrine (1:80,000; Xylocaine DENTAL, Dentsply Sirona, York, PA), the skin was removed to expose the skull. After removing the periosteum, the exposed skull was treated with bonding primer (G-CEM ONE adhesive enhancing primer, GC.dental, Tokyo, Japan). A metal plate for head fixation was attached to the skull with dental resin cement (G-CEM ONE neo, GC.dental), centering the eye within the window of the plate (Fig. 1*B*). The metal plate was secured to the skull using dental cement (G-CEM ONE neo, GC.dental). The mouse was then secured in a fixation apparatus (SGM-4; Narishige, Japan) using additional dental resin (UNIFAST II, GC.dental) (Fig. 1*C*). The plastic cap on the eyeball was removed, and the eyelashes were trimmed with ophthalmic scissors. The ophthalmic gel was rinsed off the cornea with saline. A drop of tropicamide (Mydrin M 0.4%, Santen Pharmaceutical, Osaka, Japan) was applied to induce mydriasis. After confirming successful mydriasis, a fresh layer of ophthalmic gel was applied to the cornea. A custom-made PMMA contact lens, designed to fit the mouse’s eyeball, was mounted on the cornea (Fig. 1 *D*–*F*). The key optical specifications assessed by precision interferometry (Noncontact 3D measuring instruments, NH-3SP; Mitaka Kohki Co., Ltd., Tokyo, Japan) were as follows: 1) the curvature radius was 1.65 mm, which precisely matched the mouse eyeball; 2) peak-to-valley value, reflecting the maximum deviation between the highest and lowest points on the lens surface, was 0.85 µm (design specification) and 0.78 µm (measurement), indicating minimal surface deviation and high manufacturing precision; 3) RMS value of the lens surface from the ideal design curvature was 0.20 µm (design specification) and 0.13 µm (measurement), calculated from deviations across ± 1.1 mm range from the lens center, ensuring uniform imaging quality within the retinal FOV. A glycerin reservoir, made from a cell culture dish, was secured to the fixation apparatus, sealing the space between the reservoir and the contact lens with dental impression silicone (EXAFINE injection type, GC.dental) to prevent glycerin leakage (Fig. 1*G*). The reservoir was filled with glycerin and placed under the microscope’s objective lens for imaging (Fig. 1*H*).

### In Vivo Two-Photon Imaging.

After head fixation, in vivo two-photon imaging of retinal microglia was performed on Cx3cr1^GFP/+^ mice 5 wk after STZ or vehicle injection, or 6 h after LPS injection. Two-photon images were acquired using an A1MP+ system (NIS-Elements, Nikon Instech Co., Ltd., Tokyo, Japan) with a glycerin immersion ×20 objective lens (numerical aperture [NA] 1.0, working distance 8.2 mm; CFI90 20XC Glyc, Nikon Instech Co., Ltd.) and a mode-locked Ti:sapphire Chameleon Ultra II laser (Chameleon Vision, Coherent, CA) set at 950 nm. Fluorescence emissions were collected using a GaAsP photomultiplier tube (NDD EPI unit A1-GNEN, Nikon Instech Co., Ltd.). Fluorescence was separated with two dichroic mirrors: 560-nm dichroic mirror with 500 to 550 nm (green channel for EGFP fluorescence detection) and 563 to 588 nm (red channel for tdTomato or Evans blue fluorescence detection) emission filters, and 593-nm dichroic mirror with 601 to 657 nm (magenta channel for Texas Red fluorescence detection) emission filters. Evans blue (20 mg/kg; Sigma-Aldrich, St. Louis, MO) was intraperitoneally injected to visualize retinal blood vessels (Fig. 1*I*), and DyLight 594-conjugated Isolectin B4 (0.5 mg/kg; Vector Laboratories, Burlingame, CA) was used to label vascular endothelial cells (*SI Appendix,* Fig. S1*A*).

The Z-plane (focal depth) was manually set for each mouse based on the vascular anatomy visible in the vascular imaging channel. Specifically, large vessels located in the GCL were used as anatomical landmarks, and the FOV was selected in the region between major vessels. The imaging depth was then adjusted into deeper layers to visualize intermediate vessels and capillaries, which enabled the identification of the inner plexiform layer (IPL) and OPL. The optimal Z-plane was selected at the level where microglial morphology appeared most clearly in each animal, and was kept fixed throughout each time-lapse recording. To quantify microglial morphology, we captured sequential images at a resolution of 1,024 × 1,024 pixels over a 10-min period at specific depths (scanning rate, 0.25 Hz), and repeated this procedure in different locations on the retina (up to four locations). We note that for experiments lasting less than 2 h, a single ketamine injection was used. For longer experiments (up to 4 h), ketamine/xylazine doses were reduced by one-third and supplemented with isoflurane (1.0%; Viatris, Pittsburgh, PA).

We also note that, in the initial set of experiments, two-photon microscopy and confocal microscopy (see below) were performed on separate cohorts to avoid potential artifacts in flat-mounted retinas that could arise from prior in vivo imaging. However, in the revised experiments, both imaging modalities were conducted using contralateral eyes from the same animals to minimize interanimal variability. Specifically, two-photon imaging was performed on one eye, whereas the contralateral eye was used for confocal microscopy after flat-mount preparation. This approach allowed for direct comparison of retinal microglial morphology under matched biological and experimental conditions.

### Intravitreal Viral Injection.

To image retinal ganglion cells (RGCs), Müller glia, and astrocytes, intravitreous injections of AAV2.hSyn.tdTomato (9.5 × 10^11^ vg/mL) for RGCs (*SI Appendix,* Fig. S1*B*), AAV2.GFAP.tdTomato (5.0 × 10^11^ vg/mL) for Müller glia (*SI Appendix,* Fig. S1*C*), and a combination of AAV5.GFAP.Cre.WPRE.hGH (8.8 × 10^11^ vg/mL) and AAV1.FLEX.tdTomato (1.3 × 10^12^ vg/mL) for retinal astrocytes (*SI Appendix,* Fig. S1*D*) were performed. For the intravitreous viral injection, mice were anesthetized with isoflurane, and head fixation was carried out. After disinfecting the eyeball with 10% povidone-iodine solution, an incision was made in the conjunctiva using ophthalmic scissors. The sclera at the limbus was then punctured with a custom-made tungsten needle to create a scleral window. Through this window, 2 µL of AAV was injected into the vitreous body by pressure through a glass micropipette attached to a microinjector (I-31, Narishige, Tokyo, Japan).

### Systemic Drug Administration.

Liraglutide (400 µg/kg/day; Novo Nordisk, Bagsværd, Denmark) was administered subcutaneously for 3 consecutive days to both diabetic mice 5 wk after STZ injection, and nondiabetic mice 5 wk after citrate buffer injection. Diabetic mice in the untreated group, used for the experiments in Fig. 4, received saline injections for 3 consecutive days.

### Immunohistochemistry.

Mice were deeply anesthetized with ketamine/xylazine, and transcardially perfused with 4% paraformaldehyde solution in phosphate-buffered saline (PBS). Eyes were extracted, and the retinal cups were isolated and postfixed overnight. After blocking in 1% bovine serum albumin soaked in 0.3% PBS/Triton X-100 (PBST) for 60 min at room temperature, retinal cups were immunostained overnight with primary and secondary antibodies and washed five times with 0.1% PBST. The washed retina was incised in four directions, and mounted flat on a slide glass with VECTASHIELD Mounting Medium for Fluorescence with DAPI (Vector Laboratories) for flat-mount tissue imaging. Z-stacked images (approximately 30 stacks, 1 µm intervals) were captured using an Olympus FV3000 confocal microscope (Olympus, Tokyo, Japan) with a ×40 objective (NA 0.95; Olympus) from the superficial to deep retinal vascular layers, based on previously reported methods (10, 48). Four retinal locations were imaged per flat-mount.

The following antibodies were used for staining: Goat anti-CD31 (AF3628, R&D Systems, Minneapolis, MN; 1:200), Rabbit anti-IBA1 (ab178846, Abcam, Cambridge, UK; 1:500), Rabbit anti-NG2 (AB5320, Merck Millipore, Burlington, MA; 1:200), Rat anti-CD68 (MCA1957GA, Bio-Rad, Hercules, CA; 1:400), Rat anti-CD169 (MCA884, Bio-Rad, Hercules, CA; 1:500), Guinea pig anti-P2Y12R (011-28873, Fujifilm Wako, Osaka, Japan; 1:500), Anti-rat Cy3 (AB_2340667, Jackson ImmunoResearch, West Grove, PA; 1:1,000), Anti-guinea pig Alexa Fluor 594 (A21207, Invitrogen, Carlsbad, CA; 1:1,000), Anti-rabbit Alexa Fluor 594 (ab150188, Abcam, Cambridge, UK; 1:1,000), Anti-goat Cy5 (ab6566, Abcam, Cambridge, UK; 1:1,000), and Anti-rabbit Alexa Fluor 680 (A-21076, Invitrogen, Carlsbad, CA; 1:1,000).

### Image Analysis.

Images were analyzed using ImageJ (v.1.54f; NIH, Bethesda, MD) and MATLAB (R2023b; MathWorks, Natick, MA) software packages. To correct for lateral (XY) motion artifact, we applied the ImageJ plug-in TurboReg function to two-photon images for focal plane displacement, using the vascular signal (channel 1) as a reference. Each frame was aligned to an average intensity image of the blood vessels. The same transformation matrix was then applied to the microglial channel (channel 2), ensuring accurate alignment across time. Following motion correction, we applied temporal averaging over five consecutive frames at each time point to reduce noise and enhance image quality in the microglial channel. These processing steps ensured the reliable tracking of microglial dynamics without introducing artificial artifacts. Unlike a previous cortical microglial imaging study (16), this study did not acquire Z-stacked images, but instead captured them sequentially at a fixed depth. We found that the image quality of retinal microglial processes was improved when TurboReg was applied to Z-fixed images.

Maximum intensity projection images, generated after TurboReg processing, were used for the following analyses: 1) cell density, the number of microglia in each FOV was counted using the Multi-Point Tool in ImageJ; 2) cell body size, the area of each cell body was measured using the Wand Tool; 3) territory area, the area enclosed by all the distal tips of a single microglia was measured using the Polygon Selection Tool; 4) primary process length, the primary processes of individual microglia were traced using the Simple Neurite Tracer (SNT) plug-in, and their length was measured at each time point; 5) total process length, the distal tips of each microglial processes were plotted and the X-Y coordinate measurements were conducted at each time point using the Manual Tracking plug-in; 6) cell body size change and territory area change, the cumulative absolute values of changes in cell body size and territory area over 10 min were calculated; 7) process tip movement distance, the cumulative absolute movement distances of individual process tip between consecutive frames over 10 min were measured; and 8) process tip movement area, the area enclosed by the trajectories of individual process tip over 10 min was calculated. For measurements involving multiple processes of a single microglial cell (i.e., primary process length, total process length, process tip movement distance, and process tip movement area), the values were averaged to represent the representative value of each microglial cell.

To minimize potential bias, all image analyses were conducted in a blinded manner by two authors (Hidenori Shima and Yuka Mori), who were not informed of the experimental group assignments.

### Statistics.

All statistical analyses were performed using Excel software (Microsoft, Redmond, WA), MATLAB using built-in functions, and R (version 4.4.2; using the “tidyverse”, “multcomp”, “rstatix”, “nlme”, “emmeans”, and “PMCMRplus” packages). The number of animals, the number of FOVs, and the number of microglial cells used in each experiment are specified in the figure legends and *SI Appendix,* Table S3. To assess the distribution of each dataset, normality was evaluated using the Shapiro–Wilk test when the sample size was 30 or less. For datasets with a sample size greater than 30, visual inspection with Q–Q plots and histograms was performed to assess skewness and kurtosis. If these visual inspections did not indicate extreme skewness or kurtosis, the data were assumed to be normally distributed based on the central limit theorem. For datasets that were evaluated to be normally distributed, parametric tests were applied: Welch’s *t* test for two-group comparisons and ANOVA followed by Bonferroni’s post hoc test for multigroup comparisons. Conversely, for datasets that did not meet the assumption of normality, nonparametric tests were used: Mann–Whitney U test for two-group comparisons, and Kruskal–Wallis test followed by Dunn’s multiple comparison test with Bonferroni correction or the Friedman test followed by Conover’s multiple comparison test with Bonferroni correction for multigroup comparison. Data in the figures are presented as the mean ± SEM. Statistical details, including *P*-values, are provided in the figure legends.

## Supplementary Material

Appendix 01 (PDF)

Dataset S01 (XLSX)

Movie S1. Representative movie from two-photon microscopy observations of retinal microglia (labeled with GFP) and blood vessels (labeled with Evans blue) in a Cx3cr1^GFP/+^ mouse (control). The movie captures 30 minutes of continuous imaging, demonstrating the dynamic behavior of microglial processes. The inset highlights an enlarged view of the movements of a single microglial process within the area indicated by a white rectangle.

Movie S2. Representative movies showing the dynamic movements of retinal microglial processes in control (CTL) and diabetic (DM) mice. The movies capture 10 minutes of continuous retinal imaging. Green lines indicate the trajectories connecting the initial (yellow dots) and end points (red dots) of individual microglial process tips. Yellow polygons indicate the surveillance territories of individual microglial process tips during the imaging period.

## Data Availability

This study did not generate new unique reagents, and no custom code was used. Numerical data underlying the figures have been deposited in Mendeley Data as “Transpupillary in vivo two-photon imaging reveals enhanced surveillance of retinal microglia in diabetic mice” and are available at https://doi.org/10.17632/7n95hky9zg.1 (59). All other data are included in the manuscript and/or supporting information.
