# Supplementary material for: Transpupillary in vivo two-photon imaging reveals enhanced surveillance of retinal microglia in diabetic mice
Source: Proc Natl Acad Sci U S A. 2025 Oct 8;122(41):e2426241122. doi: 10.1073/pnas.2426241122 (PMC12541322; doi:10.1073/pnas.2426241122)
Supplement: Supplementary file 1 — Appendix 01 (PDF) [file pnas.2426241122.sapp.pdf]

PNAS

# Supporting Information for

Transpupillary *in vivo* two-photon imaging reveals enhanced surveillance of retinal microglia in diabetic mice.

**Authors**:
Noriyuki Sotani [1, 2], Sentaro Kusuhara [2, *], Ryuto Nishisho [2], Hiroto Kuno [1], Hidenori Shima [1], Koichiro Haruwaka [3], Yuka Mori [1], Maya Kishi [2], Tomoyuki Furuyashiki [4], Kenta Kobayashi [5], Hiroaki Wake [6], Toru Takumi [1], Makoto Nakamura [2], Yoshihisa Tachibana [1, *]

**Corresponding authors**:
Dr. Yoshihisa Tachibana
E-mail: yoshi@med.kobe-u.ac.jp

Dr. Sentaro Kusuhara
E-mail: kusu@med.kobe-u.ac.jp

**This PDF file includes:**

Figures S1 to S10
Tables S1 to S3
Legends for Movies S1 to S2
Validation of two-photon microscopy data (conversion from pixel to micrometer units)

**Other supporting materials for this manuscript include the following:**

Movies S1 to S2

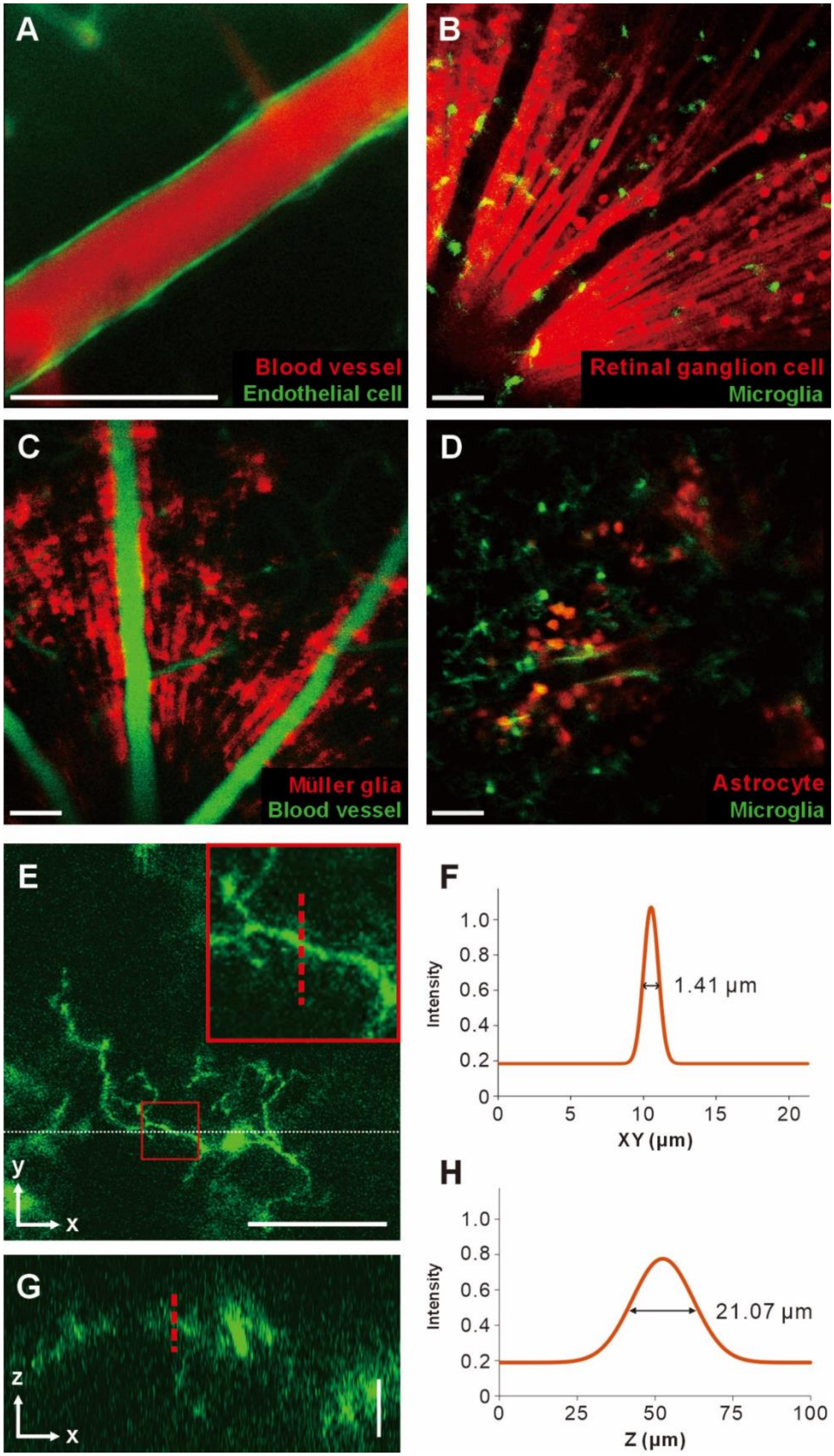

A
Blood vessel
Endothelial cell
B
Retinal ganglion cell
Microglia
C
Müller glia
Blood vessel
D
Astrocyte
Microglia
E
y
x
F
Intensity
1.41 µm
XY (µm)
G
z
x
H
Intensity
21.07 µm
Z (µm)

**Fig. S1.** Representative images of two-photon microscopy observation of retinal components. (*A*) Blood vessels (red fluorescence labeled with Evans blue) and vascular endothelial cells (green fluorescence labeled with Isolectin B4-Fluorescein). (*B*) Retinal ganglion cells (labeled with tdTomato) and microglia (labeled with GFP). (*C*) Müller glia (labeled with tdTomato) and blood vessels (labeled with fluorescein). (*D*) Astrocytes (labeled with tdTomato) and microglia (labeled with GFP). Scale bars, 50 µm. (*E-H*) Estimation of imaging resolution using the microglial processes captured by our two-photon imaging system. (*E*) Maximum intensity projection images (x-y plane) of microglia. The inset, indicated by a red square, shows a magnified view of a microglial process. Scale bar, 50 µm. (F) The transverse cross-section of point objects on the microglial process along the red dashed line in (*E*) was used to estimate the lateral imaging resolution. (*G*) Orthogonal slice (x-z plane) along the white dashed line indicated in (*E*). (*H*) The axial cross-section of point objects on the microglial process (red dashed line in *G*) was used to estimate the axial imaging resolution. The full width at half maximum (FWHM) of the lateral and axial resolutions for this representative microglial process was measured to be 1.41 µm and 21.07 µm, respectively. Lateral resolutions from two additional measured processes were 1.20 µm and 1.52 µm, and the corresponding axial resolutions were 16.36 and 21.95 µm. The conversion from pixel to micrometer units was applied according to the procedure detailed in the final section of this Supporting Information. Scale bar, 100 µm.

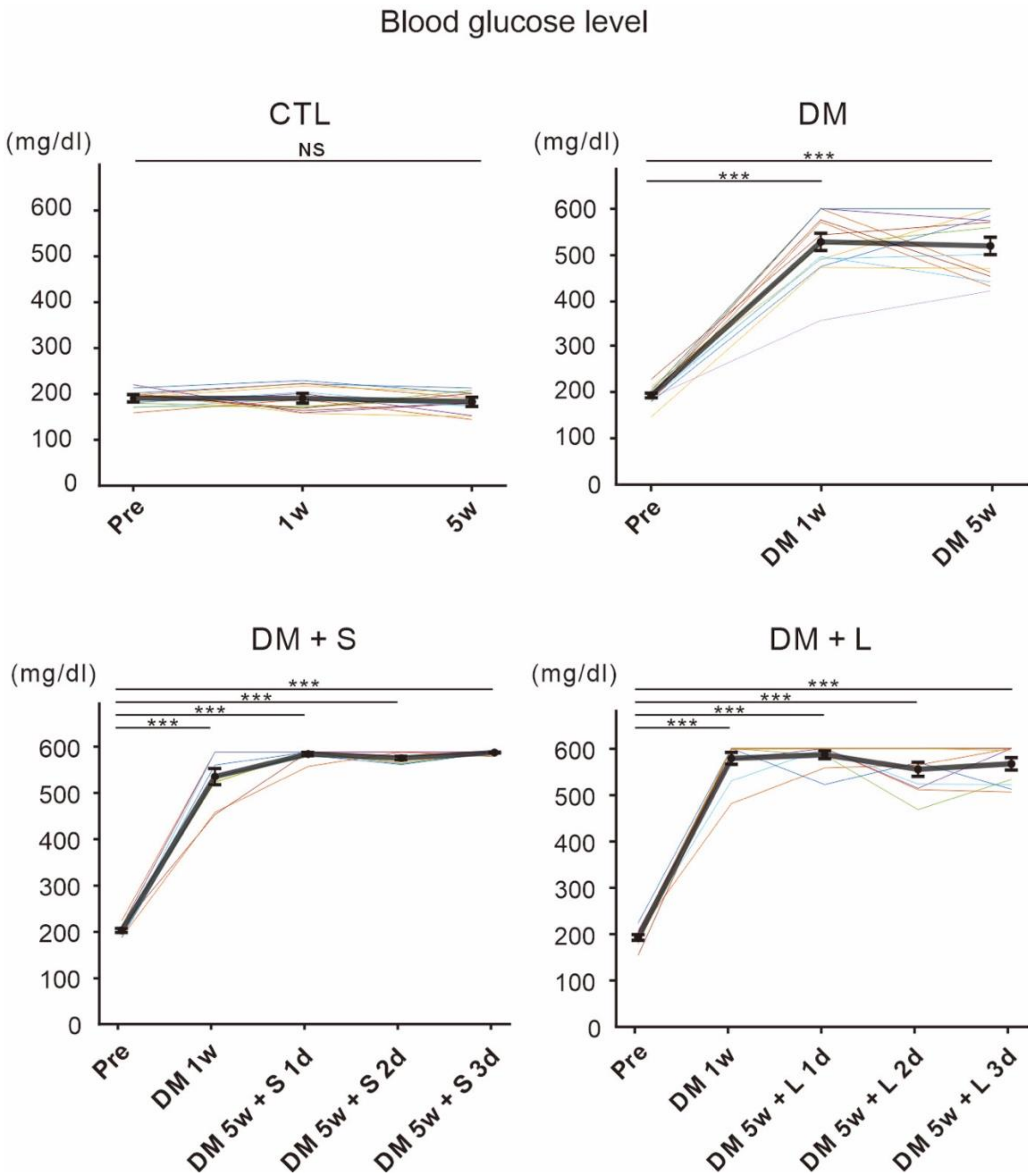


**Fig. S2.** Time course of blood glucose levels in four experimental groups: 1) controls (CTL; 14 mice), 2) streptozotocin (STZ)-treated diabetic mice (DM; 14 mice), 3) STZ-treated DM mice administered saline for three days (DM + S; nine mice), and 4) STZ-treated DM mice administered liraglutide for three days (DM + L; 10 mice). Blood glucose levels were monitored throughout the experimental period to evaluate the effects of STZ injection and the subsequent administration of saline or liraglutide. NS, not significant; ***$p < 0.001$ (Friedman test followed by Conover's multiple comparison test with Bonferroni correction). Data are presented as mean ± SEM.

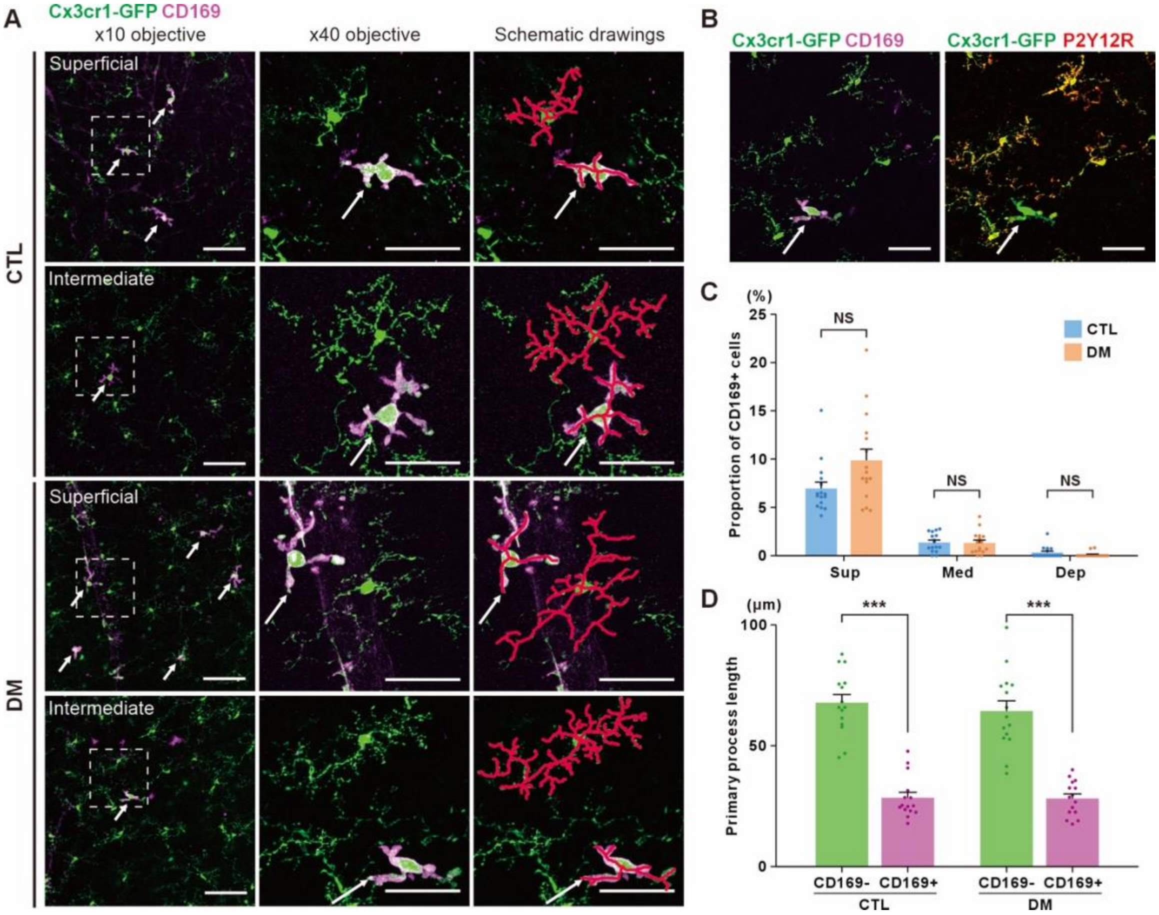


**Fig. S3.** Identification of GFP-positive cells as resident microglia or infiltrating macrophages in the retina of Cx3cr1 $^{GFP/+}$ mice. (*A*) Confocal images showing GFP-positive cells (green) and CD169-positive cells (magenta) in the superficial (ganglion cell layer, GCL; upper panels) and intermediate (inner plexiform layer, IPL; lower panels) layers of control (CTL) and diabetic (DM) retinas. From left to right: low-magnification photomicrographs acquired with a ×10 objective lens (scale bars: 100 μm), high-magnification images of the dashed-square regions acquired with a ×40 objective lens (scale bars: 50 μm), and schematic drawings of CD169-positive cells from the x40 images. CD169-positive cells (indicated by white arrows) exhibited an ameboid-shaped morphology with shorter primary processes and a highly polarized appearance, in contrast to the ramified morphology of CD169-negative cells. (*B*) Confocal images (x40 objective lens; scale bars: 50 μm) showing triple labeling of GFP-positive cells (green), CD169-positive cells (magenta), and P2Y12R-positive cells (red) in the retina. P2Y12R-positive cells and CD169-positive cells were spatially segregated, supporting their identification as infiltrating macrophages and resident microglia, respectively. (*C*) Quantification of the proportion of CD169-positive cells among all GFP-positive cells across the superficial, intermediate, and deep retinal layers. Each data point represents a single field of view (FOV). A total of 16 FOVs per layer were analyzed from four mice for each group (CTL and DM). NS, not significant (Kruskal-Wallis test followed by Dunn's multiple comparison test with Bonferroni correction). (*D*) Quantitative data on the primary process length of CD-169 positive versus CD-169 negative cells in CTL and DM retinas. A total of 15 microglial cells were analyzed from four mice for each group. ***$p < 0.001$ (Kruskal-Wallis test followed by Dunn's multiple comparison test with Bonferroni correction). Data are presented as mean ± SEM.

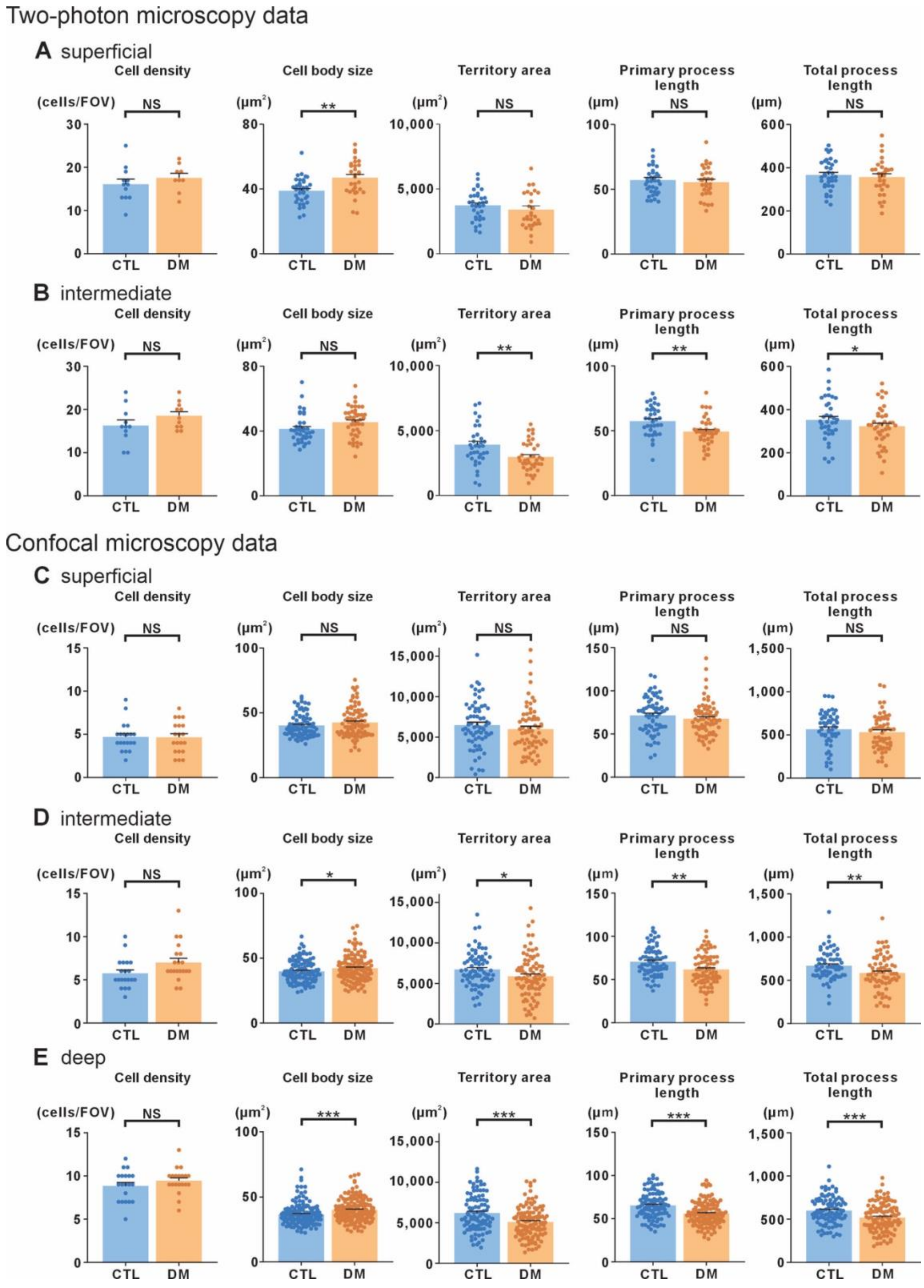


**Fig. S4.** Layer-specific analysis of static morphological changes in retinal microglia of diabetic mice. (*A*-*B*) Quantitative data on microglial morphology based on two-photon microscopy images from the superficial (*A*) and intermediate (*B*) retinal layers of control (CTL) and diabetic (DM)

mice. Five morphological parameters were evaluated: cell density, cell body size, territory area, primary process length, and total process length. For the analysis of cell density in the superficial layer, 12 and 9 field of views (FOVs) were analyzed for the six CTL and five DM mice, respectively. In the intermediate layer, 11 and 11 FOVs were analyzed for the six CTL and eight DM mice, respectively. For the remaining parameters in the superficial layer, 35 and 30 microglial cells were analyzed for the six CTL and five DM mice, respectively. In the intermediate layer, 37 and 42 microglial cells were analyzed for six CTL and eight DM mice, respectively. (*C*-*E*) Quantitative data on microglial morphology based on confocal microscopy images from the superficial (*C*), intermediate (*D*), and deep (*E*) retinal layers of CTL and DM mice, presented in the same format as (*A*-*B*). Data were sampled from five mice for each group. For cell density, 20 FOVs were analyzed in each group across all layers. For cell body size, 85 and 88 microglial cells were analyzed in the superficial layer, 110 and 128 cells were analyzed in the intermediate layer, and 155 and 165 cells were analyzed in the deep layer for CTL and DM mice, respectively. For the remaining parameters, 66 and 68 microglial cells were analyzed in the superficial layer, 76 and 85 cells were analyzed in the intermediate layer, and 98 and 114 cells were analyzed in the deep layer for CTL and DM mice, respectively. NS, not significant; $^*p < 0.05$; $^{**}p < 0.01$; $^{***}p < 0.001$ (Welch's t-test; Mann-Whitney U test was applied only for cell density in (*C*-*E*)). Data are presented as mean ± SEM.

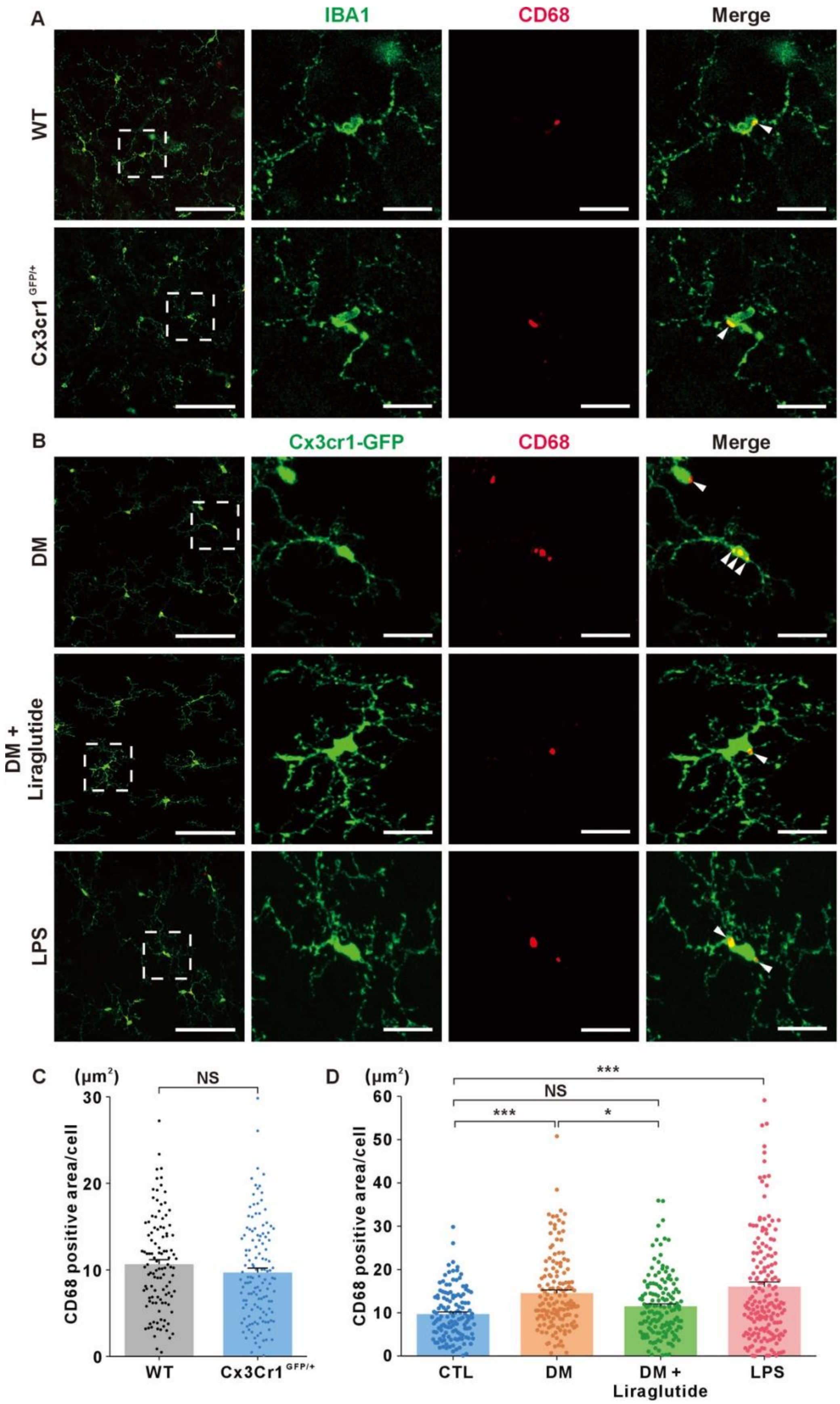
A
IBA1
CD68
Merge
WT
Cx3cr1 GFP/+
B
Cx3cr1-GFP
CD68
Merge
DM
DM + Liraglutide
LPS
C
(μm²)
NS
CD68 positive area/cell
30
20
10
0
WT
Cx3Cr1 GFP/+
D
(μm²)
***
NS
***
*
60
50
40
30
20
10
0
CD68 positive area/cell
CTL
DM
DM + Liraglutide
LPS

**Fig. S5.** Differential microglial activation revealed by CD68 immunoreactivity under genetic and disease-associated conditions. (*A*) Representative confocal images of microglia in the intermediate layer of the retina immunostained for IBA1 (green) and CD68 (red) in wild-type Cx3cr1$^{GFP/GFP}$ (WT) and heterozygous Cx3cr1$^{GFP/+}$ mice. Merged images show co-localization of CD68 with microglial cell bodies or processes, indicating lysosomal activation. The leftmost panels show low-magnification photomicrographs acquired with a x10 objective lens (scale bars: 100 μm). The right panels display high-magnification images of the dashed-square regions from the leftmost panels, acquired with a x40 objective lens (scale bars: 20 μm). Arrowheads indicate CD68-positive areas. (*B*) CD68 immunoreactivity in diabetic mice (DM), DM mice treated with liraglutide (DM + Liraglutide), and lipopolysaccharide (LPS)-injected mice, shown as in (*A*), except that GFP (green) was used to visualize microglia. (*C*) Quantification of CD68-positive area normalized to microglial territory area in WT and Cx3cr1$^{GFP/+}$ mice. A total of 111 and 130 microglial cells were analyzed from five mice in the WT and Cx3cr1$^{GFP/+}$ groups, respectively. CD68 expression did not differ significantly between the two groups. NS, not significant (Welch's t-test). (*D*) Quantification of CD68-positive area in the CTL, DM, DM + Liraglutide, and LPS groups. A total of 143, 150, and 173 microglial cells from five mice were analyzed in the DM, DM + Lira, and LPS groups, respectively. The same dataset was used for the Cx3cr1$^{GFP/+}$ group in (*C*) and the CTL group in (*D*). For (*C*) and (*D*), the images were obtained from all retinal layers (superficial, intermediate, and deep layers). NS, not significant; *$p < 0.05$; ***$p < 0.001$ (one-way ANOVA followed by Bonferroni's post hoc test). Data are presented as mean ± SEM.

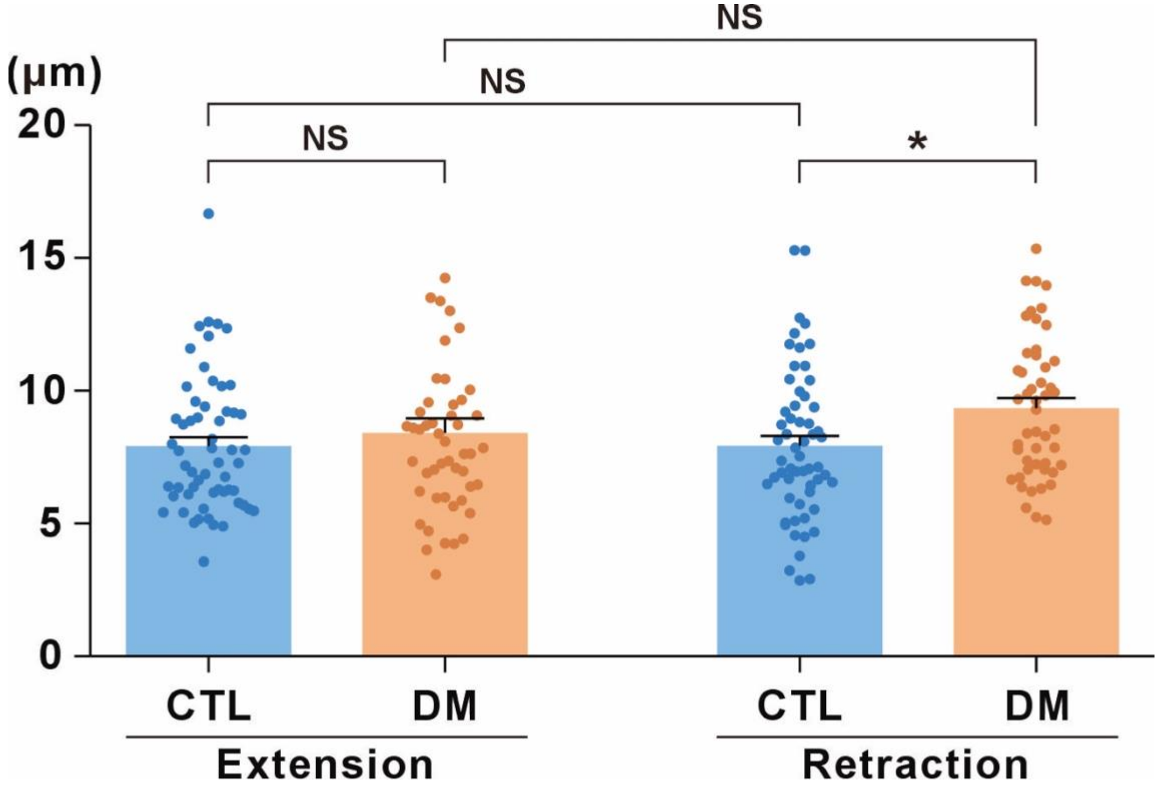


**Fig. S6.** Quantitative analysis of cumulative extension and retraction lengths (speeds) of retinal microglial process tips in control (CTL) and diabetic (DM) mice, based on two-photon microscopy images. A total of 58 and 49 microglial cells from seven mice were analyzed in the CTL and DM groups, respectively. The extension length (speed) represents the total distance a process tip extended, whereas the retraction length (speed) represents the total distance a process tip retracted during a 10-minute observation period. NS, not significant; *$p < 0.05$ (two-way ANOVA followed by Bonferroni's post hoc test). Data are presented as mean ± SEM.

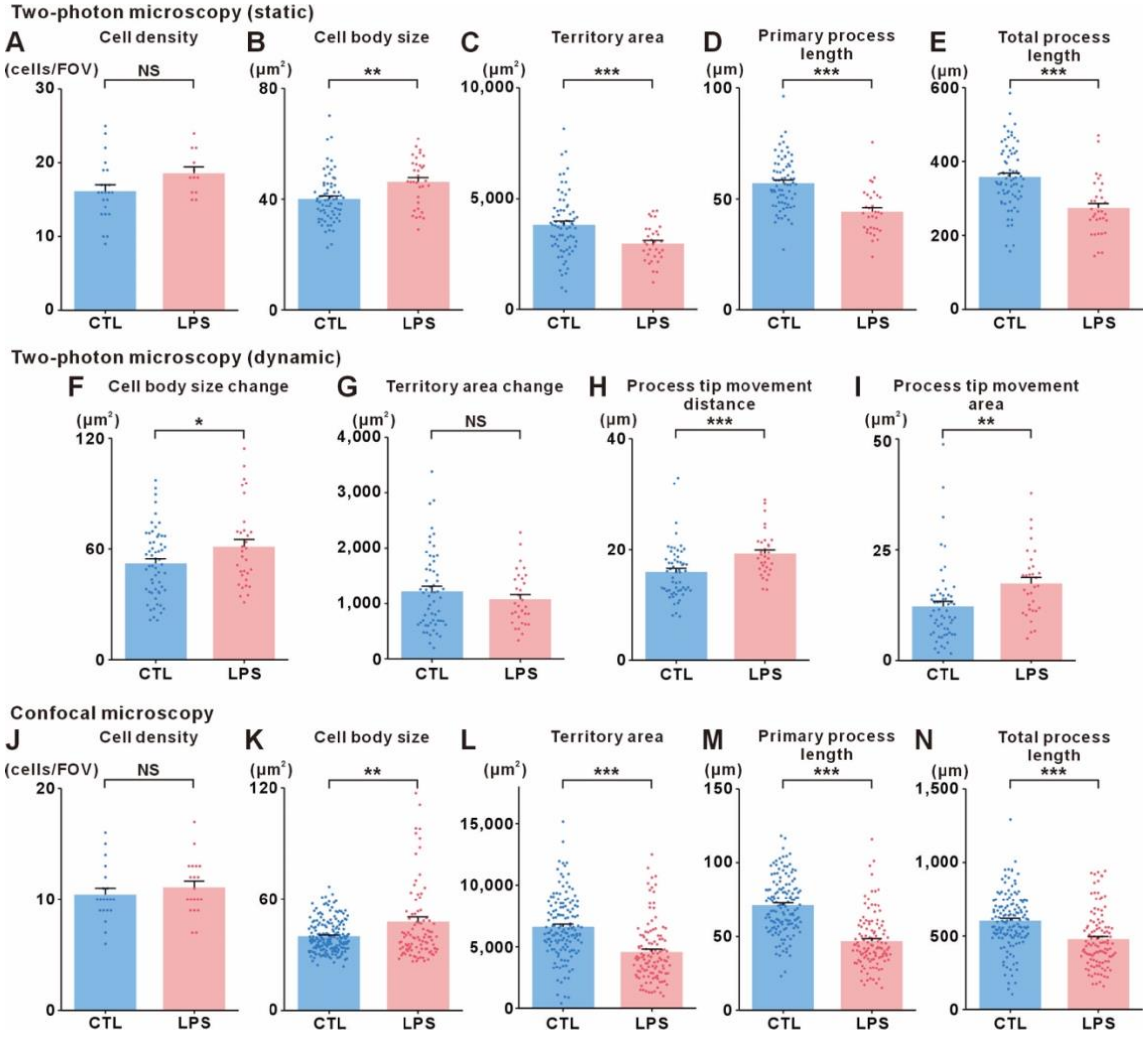


**Fig. S7.** Static and dynamic analysis of retinal microglia in mice injected intraperitoneally with lipopolysaccharide (LPS). (*A*-*E*) Quantitative data on microglial morphology in control (CTL) and LPS-injected mice based on two-photon microscopy images. (*A*) Density of microglial cells per field of view (FOV). A total of 23 and 12 FOVs were analyzed for nine CTL and five LPS mice, respectively. (*B*-*E*) Cell body size (*B*), territory area (*C*), primary process length (*D*), and total process length (*E*) of individual microglial cells. For these parameters, 72 and 33 microglial cells were analyzed for the nine CTL and five LPS mice, respectively. (*F*-*I*) Quantitative data showing dynamic changes in microglial morphology in CTL and LPS mice, based on sequential (10-minute) two-photon microscopy imaging. The data were obtained from the summation of changes over 10 minutes in cell body size (*F*), territory area occupied by all processes of a single microglial cell (*G*), movement distance of process tips (*H*), and surveillance territory area of process tips (*I*). For each parameter, 58 and 39 microglial cells were analyzed for the seven CTL and five LPS mice, respectively. The imaging protocol and quantification method for (*F*-*I*) were the same as described in Fig. 3. (*J*-*N*) Quantitative data on microglial morphology in CTL and LPS mice based on confocal microscopy images of flat-mounted retinas. (*J*) Density of microglial cells per field of view (FOV). A total of 20 FOVs from five mice were analyzed for each group. (*K*-*N*) Cell body size (*K*), territory area (*L*), primary process length (*M*), and total process length (*N*) of individual microglial cells. For these parameters, 195 and 101 microglial cells (*K*), and 142 and 114 microglial cells (*L*-*N*) from five mice were analyzed in the CTL and LPS groups, respectively. Two-photon and confocal images were sampled from the superficial and intermediate layers in

the retina**.** The datasets for the CTL group were the same as those used in Fig. 3 of the main text. NS, not significant; *$p < 0.05$; **$p < 0.01$; ***$p < 0.001$ (Welch’s t-test for all datasets). Data are presented as mean ± SEM.

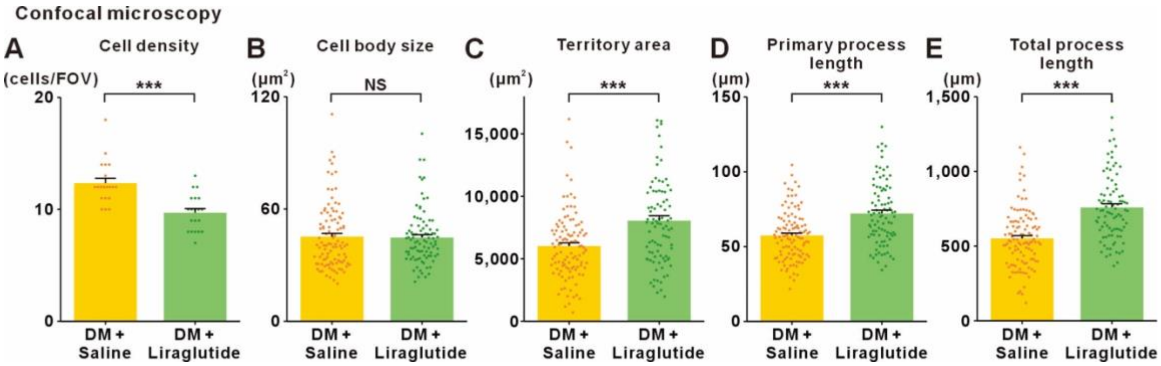


**Fig. S8.** Static analysis of morphological changes in retinal microglia of diabetic mice treated with liraglutide. (*A*-*E*) Quantitative data on microglial morphology in diabetic mice treated with saline (DM + Saline) or liraglutide (DM + Liraglutide), based on confocal microscopy images of flat-mounted retinas. (*A*) Density of microglial cells per field of view (FOV). A total of 20 FOVs from five mice were analyzed for each group. ***p < 0.001 (Mann-Whitney U test). (*B*-*E*) Cell body size (*B*), territory area (*C*), primary process length (*D*), and total process length (*E*) of individual microglial cells. For these parameters, 104 and 91 microglial cells (*B*), and 111 and 91 microglial cells (*C*-*E*) from five mice were analyzed in the DM + Saline and DM + Liraglutide groups, respectively. Confocal images were sampled from the superficial and intermediate layers in the retina**.** NS, not significant; ***p < 0.001 (Welch's t-test). Data are presented as mean ± SEM.

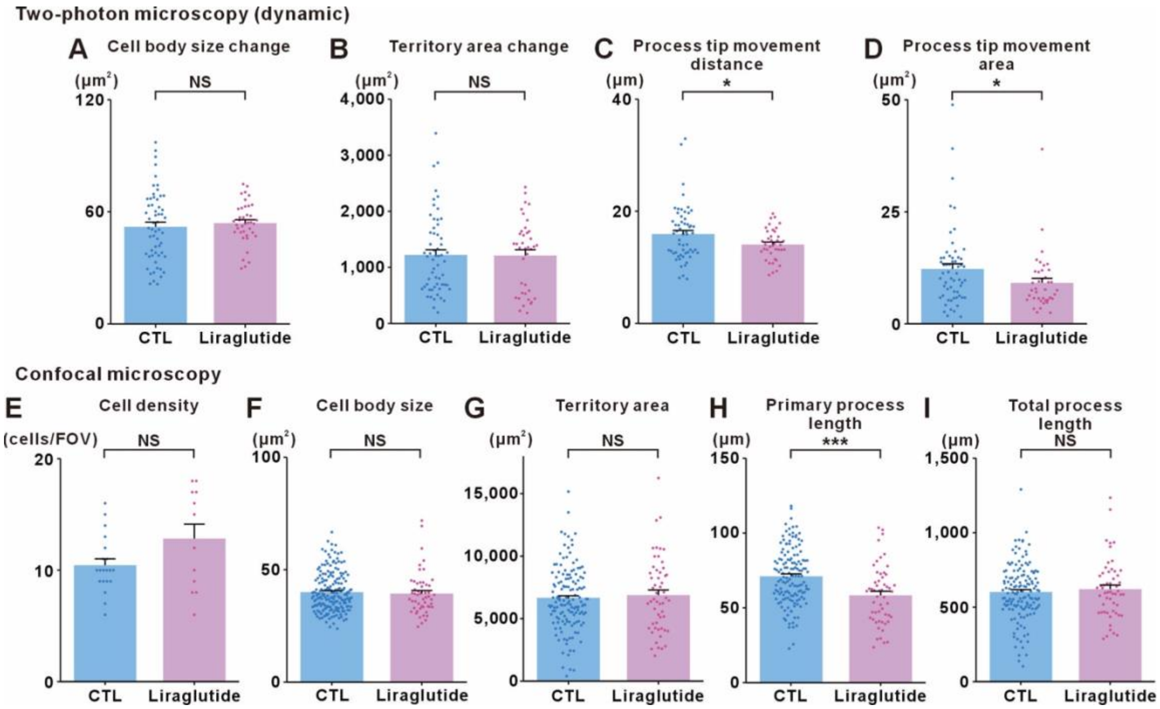


**Fig. S9.** Effects of liraglutide on retinal microglia in non-diabetic mice. (*A*-*D*) Quantitative data showing dynamic changes in microglial morphology in untreated (control, CTL) and liraglutide-treated (Liraglutide) non-diabetic mice, based on sequential (10-minute) two-photon microscopy imaging. The data were obtained from the summation of changes over 10 minutes in cell body size (*A*), territory area occupied by all processes of a single microglial cell (*B*), movement distance of process tips (*C*), and surveillance territory area of process tips (*D*). For these parameters, 58 and 39 microglial cells were analyzed from five mice in the CTL and Liraglutide groups, respectively. The imaging protocol and quantification method for (*A*-*D*) were the same as described in Fig. 3. (*E*-*I*) Quantitative data on microglial morphology in untreated and liraglutide-treated non-diabetic mice, based on confocal microscopy images of flat-mounted retinas. (*E*) Density of microglial cells per field of view (FOV). A total of 20 and 12 FOVs were analyzed for the five CTL and three Liraglutide mice, respectively. (*F*-*I*) Cell body size (*F*), territory area (*G*), primary process length (*H*), and total process length (*I*) of individual microglial cells. For these parameters, 195 and 52 microglial cells (*F*), and 142 and 54 microglial cells (*G*-*I*) were analyzed for the five CTL and three Liraglutide mice, respectively. Two-photon and confocal images were sampled from the superficial and intermediate layers in the retina. The datasets for the CTL group were the same as those used in Fig. 3 of the main text. NS, not significant; *p < 0.05; ***p < 0.001 (Welch's t-test for all datasets). Data are presented as mean ± SEM.

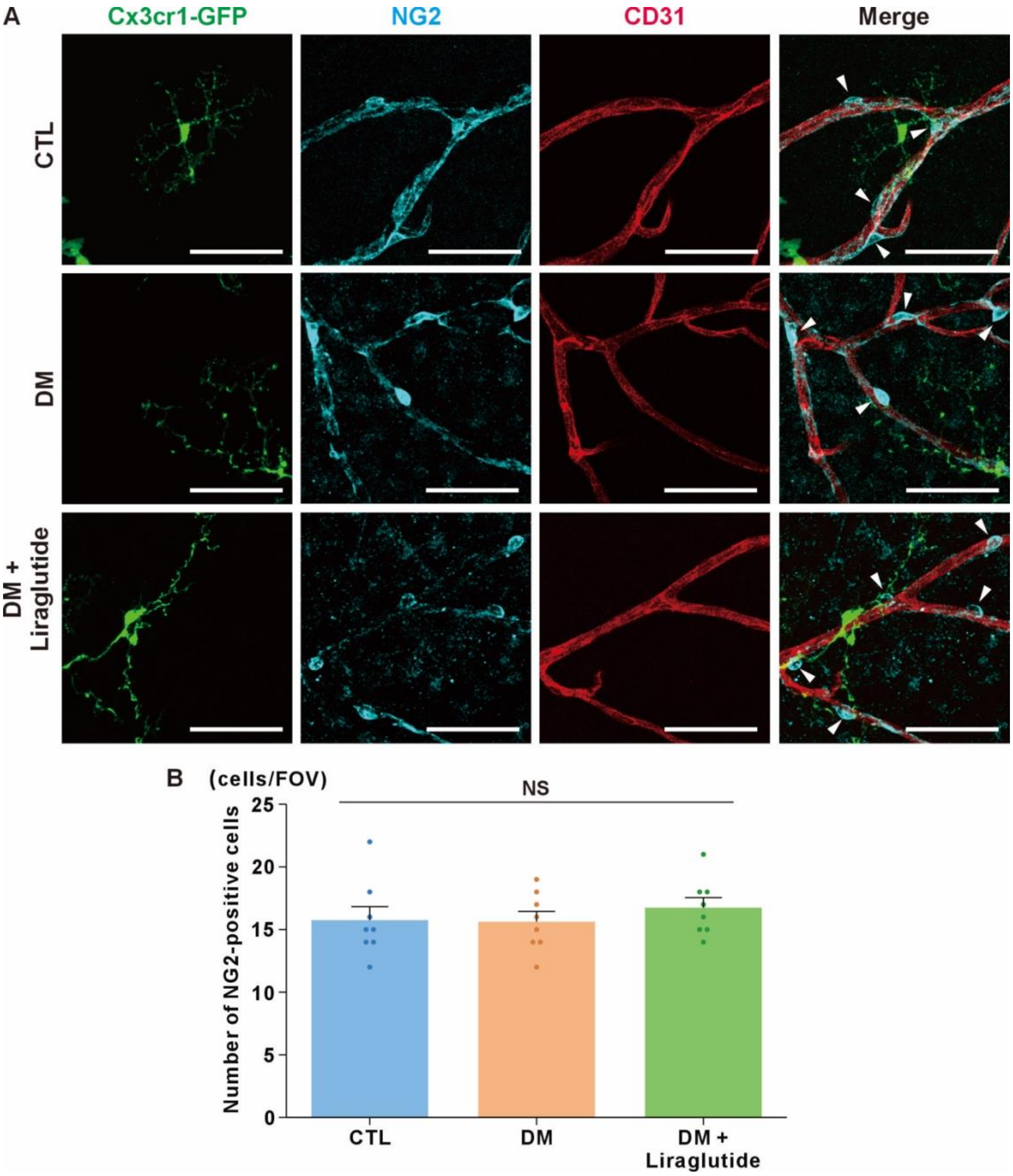


**Fig. S10.** Evaluation of pericytes surrounding blood vessels in the retina. (*A*) Representative confocal images of retinal vasculature immunostained for CX3CR1-GFP (microglial marker, green), NG2 (pericyte marker, cyan), and CD31 (endothelial cell marker, red) in control (CTL), diabetic (DM), and liraglutide-treated diabetic (DM + Liraglutide) mice. Merged images are shown in the right column. Arrowheads indicate pericytes in contact with blood vessels. Scale bars, 50 μm. (*B*) Quantification of pericyte density in the field of view (FOV) around retinal vasculature in the superficial layer. A total of eight FOVs from two mice were analyzed for each group. Statistical analysis was performed using one-way ANOVA, which revealed no significant differences in pericyte density among the three groups. Data are presented as mean ± SEM.

| | | |
|---|---|---|
| **Virus strains** | | |
| $AAV_1$-FLEX-tdTomato | Addgene | Cat# 28306-AAV1 |
| $AAV_2$-hSyn-tdTomato | Kobayashi Lab | N/A |
| $AAV_2$-GFAP-tdTomato | Kobayashi Lab | N/A |
| $AAV_5$-GFAP-Cre | Addgene | Cat# 105550-AAV5 |
| **Antibodies and chemicals** | | |
| Goat Anti-CD31 | R&D Systems | Cat# AF3628 |
| Rabbit Anti-Iba1 | Abcam | Cat# ab178846 |
| Rabbit Anti-NG2 Chondroitin Sulfate Proteoglycan Antibody | Sigma-Aldrich | Cat# AB5320 |
| Rat Anti-CD68 | Biorad | Cat# MCA1957GA |
| Rat Anti-CD169 | Biorad | Cat# MCA884 |
| Guinea pig Anti-P2Y12 | FUJIFILM Wako Pure Chemical Corporation | Cat# 011-28873 |
| Donkey Anti-Rat IgG (H+L) Cy™3 AffiniPure® | Jackson ImmunoResearch | RRID: AB_2340667 |
| Goat Anti-Guinea pig IgG H&L (Alexa Fluor® 594) | Abcam | Cat# ab150188 |
| Donkey Anti-Rabbit IgG (H+L) (Alexa Fluor™ 594) | Invitrogen | Cat# A-21207 |
| Donkey Anti-Goat IgG H&L (Cy5) | Abcam | Cat# ab6566 |
| Goat Anti-Rabbit IgG (H+L) (Alexa Fluor™ 680) | Invitrogen | Cat # A-21076 |
| UNIFAST Ⅱ | GC.dental | N/A |
| EXAFINE injection type | GC.dental | N/A |
| G-CEM ONE neo | GC.dental | N/A |
| G-CEM ONE adhesive enhancing primer | GC.dental | N/A |

| *Continued* | | |
|---|---|---|
| **Antibodies and chemicals** | | |
| Scopisol | Senju Pharmaceutical | N/A |
| Tropicamide | Santen Pharmaceutical | N/A |
| Streptozotocin | Nacalai Tesque | Cat# 32238-91 |
| Evans blue | Sigma-Aldrich | Cat# E2129 |
| Griffonia Simplicifolia Lectin I Isolectin B4, Fluorescein | Vector Laboratories | Cat# FL-1201-.5 |
| Fluorescein | Novartis Pharma K.K. | CAS: 2321-07-5 |
| Lipopolysaccharides from Escherichia coli O111:B4 | Sigma-Aldrich | CAS: 93572-42-0 |
| Liraglutide | Novo Nordisk | CAS: 204656-20-2 |
| VECTASHIELD Mounting Medium for Fluorescence with DAPI | Vector Laboratories | Cat# H-1200 |
| Ketamine | Daiichi Sankyo | N/A |
| Xylazine | Elanco Japan | N/A |
| Isoflurane | Viatris | N/A |
| Lidocaine | Dentsply Sirona | N/A |
| **Experimental models: Organisms/strains** | | |
| Mouse: C57BL6/J | Japan SLC | C57BL/6JJmsSlc |
| Mouse: B6.129P-Cx3cr1tm1Litt/J | The Jackson Laboratory | JAX: 005582 |
| **Software and algorithms** | | |
| Matlab R2023b | MathWorks | RRID: SCR_001622 |
| ImageJ v.1.54f | National Institute of Health | RRID: SCR_003070 |
| R 4.4.2 | Comprehensive R Archive Network | N/A |

**Table S1.** Key reagents

Two-photon microcopy data (control vs. STZ-induced diabetic mice in Figures 2&3)

| Group | Pre injection | | 1 week after injection | | 5 weeks after injection | |
|---|---|---|---|---|---|---|
| | Weight, g | Glucose, mg/dl | Weight, g | Glucose, mg/dl | Weight, g | Glucose, mg/dl |
| CTL (n = 9) | 25.14 ± 0.41 | 188.56 ± 5.00 | 27.08 ± 0.32 | 184.11 ± 7.78 | 29.02 ± 0.60 | 181.11 ± 6.86 |
| DM (n = 9) | 23.63 ± 0.43 | 193.78 ± 7.30 | 24.63 ± 0.57 | 517.33 ± 25.47 | 23.79 ± 0.53 | 512.89 ± 23.53 |
| | NS | NS | ** | *** | *** | *** |

Confocal microscopy data (control vs. STZ-induced diabetic mice in Figure 2)

| Group | Pre injection | | 1 week after injection | | 5 weeks after injection | |
|---|---|---|---|---|---|---|
| | Weight, g | Glucose, mg/dl | Weight, g | Glucose, mg/dl | Weight, g | Glucose, mg/dl |
| CTL (n = 10) | 25.01 ± 0.43 | 190.50 ± 5.52 | 25.96 ± 0.50 | 191.40 ± 8.07 | 28.75 ± 0.57 | 179.80 ± 7.58 |
| DM (n = 10) | 24.63 ± 0.51 | 189.60 ± 5.84 | 24.66 ± 0.50 | 515.20 ± 23.81 | 24.36 ± 0.57 | 505.60 ± 21.45 |
| | NS | NS | NS | *** | *** | *** |

Two-photon microcopy data (saline injected vs. liraglutide-injected diabetic mice in Figure 4)

| Group | Pre injection | | 1 week after injection | | 5 weeks after injection | |
|---|---|---|---|---|---|---|
| | Weight, g | Glucose, mg/dl | Weight, g | Glucose, mg/dl | Weight, g | Glucose, mg/dl |
| DM+Saline (n = 5) | 24.18 ± 0.87 | 211.20 ± 4.78 | 24.76 ± 0.71 | 567.40 ± 15.01 | 24.22 ± 1.25 | 600.00 ± 0.00 |
| DM+Liraglutide (n = 5) | 24.16 ± 0.57 | 192.20 ± 5.06 | 23.82 ± 1.21 | 571.40 ± 23.07 | 23.26 ± 1.59 | 588.60 ± 8.18 |
| | NS | NS | NS | NS | NS | NS |

Confocal microscopy data (saline injected vs. liraglutide-injected diabetic mice in Figure S8)

| Group | Pre injection | | 1 week after injection | | 5 weeks after injection | |
|---|---|---|---|---|---|---|
| | Weight, g | Glucose, mg/dl | Weight, g | Glucose, mg/dl | Weight, g | Glucose, mg/dl |
| DM+Saline (n = 5) | 22.10 ± 0.25 | 197.80 ± 6.24 | 23.02 ± 0.19 | 521.20 ± 26.09 | 22.22 ± 0.34 | 592.20 ± 6.20 |
| DM+Liraglutide (n = 5) | 21.88 ± 0.79 | 192.20 ± 11.84 | 21.34 ± 1.46 | 585.60 ± 13.91 | 21.60 ± 1.19 | 584.40 ± 15.60 |
| | NS | NS | NS | NS | NS | NS |

**Table S2.** Blood glucose levels of mice in all experimental groups were measured in the morning using a portable glucometer. We note that the maximum blood glucose level was 600 (mg/dL). NS, not significant; **$p < 0.01$; ***$p < 0.001$ (repeated-measures two-way ANOVA followed by Bonferroni's post hoc test). Data are presented as mean ± SEM.

| Figure | Unit | Group | Mean ± SEM (per cell) | Mean ± SEM (per FOV) | Mean ± SEM (per animal) | TEST |
|---|---|---|---|---|---|---|
| Fig. 2B | cells/FOV | CTL<br>DM | | 16.17 ± 0.86 (n = 23)<br>18.10 ± 0.68 (n = 20)<br>NS | 16.48 ± 1.08 (n = 9)<br>18.09 ± 0.68 (n = 9)<br>NS | Welch's T test |
| Fig. 2C | µm$^2$ | CTL<br>DM | 40.18 ± 1.01 (n = 72)<br>46.05 ± 1.17 (n = 72)<br>*** | | 39.08 ± 1.56 (n = 9)<br>46.46 ± 1.25 (n = 9)<br>** | Welch's T test |
| Fig. 2D | µm$^2$ | CTL<br>DM | 3818.49 ± 166.10 (n = 72)<br>3117.57 ± 157.79 (n = 72)<br>** | | 3772.73 ± 246.02 (n = 9)<br>3156.82 ± 267.27 (n = 9)<br>NS | Welch's T test |
| Fig. 2E | µm | CTL<br>DM | 57.14 ± 1.38 (n = 72)<br>51.77 ± 1.36 (n = 72)<br>** | | 56.43 ± 1.71 (n = 9)<br>52.32 ± 2.25 (n = 9)<br>NS | Welch's T test |
| Fig. 2F | µm | CTL<br>DM | 359.84 ± 10.02 (n = 72)<br>326.35 ± 9.99 (n = 72)<br>* | | 366.80 ± 15.92 (n = 9)<br>330.09 ± 14.68 (n = 9)<br>NS | Welch's T test |
| Fig. 2I | cells/FOV | CTL<br>DM | | 10.45 ± 0.56 (n = 20)<br>11.65 ± 0.63 (n = 20)<br>NS | 10.45 ± 0.58 (n = 5)<br>11.65 ± 0.69 (n = 5)<br>NS | Mann-Whitney U test |
| Fig. 2J | µm$^2$ | CTL<br>DM | 40.03 ± 0.61 (n = 195)<br>42.37 ± 0.73 (n = 216)<br>* | | 39.92 ± 1.82 (n = 5)<br>42.51 ± 1.85 (n = 5)<br>NS | Welch's T test |
| Fig. 2K | µm$^2$ | CTL<br>DM | 6619.63 ± 211.69 (n = 142)<br>5931.87 ± 222.52 (n = 153)<br>* | | 6585.54 ± 296.44 (n = 5)<br>5955.62 ± 253.40 (n = 5)<br>NS | Welch's T test |
| Fig. 2L | µm | CTL<br>DM | 71.00 ± 1.57 (n = 142)<br>64.29 ± 1.52 (n = 153)<br>** | | 70.91 ± 1.72 (n = 5)<br>64.12 ± 1.41 (n = 5)<br>* | Welch's T test |
| Fig. 2M | µm | CTL<br>DM | 601.91 ± 15.65 (n = 142)<br>538.46 ± 16.42 (n = 153)<br>** | | 597.61 ± 30.33 (n = 5)<br>532.17 ± 35.09 (n = 5)<br>NS | Welch's T test |
| Fig. 3E | µm$^2$ | CTL<br>DM | 52.06 ± 2.47 (n = 58)<br>58.23 ± 2.01 (n = 49)<br>NS | | 51.55 ± 2.74 (n = 7)<br>59.18 ± 2.25 (n = 7)<br>NS | Welch's T test |
| Fig. 3F | µm$^2$ | CTL<br>DM | 1225.38 ± 92.01 (n = 58)<br>1689.26 ± 197.73 (n = 49)<br>* | | 1213.26 ± 185.27 (n = 7)<br>1548.24 ± 318.40 (n = 7)<br>NS | Welch's T test |
| Fig. 3G | µm | CTL<br>DM | 15.96 ± 0.64 (n = 58)<br>17.91 ± 0.54 (n = 49)<br>* | | 15.81 ± 1.25 (n = 7)<br>17.64 ± 0.76 (n = 7)<br>NS | Welch's T test |
| Fig. 3H | µm$^2$ | CTL<br>DM | 12.27 ± 1.14 (n = 58)<br>15.75 ± 1.18 (n = 49)<br>* | | 11.99 ± 2.15 (n = 7)<br>15.89 ± 1.78 (n = 7)<br>NS | Welch's T test |
| Fig. 4B | µm$^2$ | DM + S<br>DM + L | 53.83 ± 1.90 (n = 36)<br>54.41 ± 1.67 (n = 39)<br>NS | | 53.91 ± 1.50 (n = 5)<br>51.56 ± 1.93 (n = 5)<br>NS | Welch's T test |
| Fig. 4C | µm$^2$ | DM + S<br>DM + L | 1725.87 ± 103.75 (n = 36)<br>1247.23 ± 91.54 (n = 39)<br>*** | | 1734.83 ± 92.87 (n = 5)<br>1260.27 ± 188.69 (n = 5)<br>* | Welch's T test |
| Fig. 4D | µm | DM + S<br>DM + L | 17.45 ± 0.70 (n = 36)<br>13.44 ± 0.36 (n = 39)<br>*** | | 17.47 ± 1.15 (n = 5)<br>13.48 ± 0.68 (n = 5)<br>* | Welch's T test |
| Fig. 4E | µm$^2$ | DM + S<br>DM + L | 15.71 ± 1.41 (n = 36)<br>6.77 ± 0.44 (n = 39)<br>*** | | 15.69 ± 2.31 (n = 5)<br>6.77 ± 0.65 (n = 5)<br>* | Welch's T test |
| Fig. S2<br>(Upper left, CTL) | mg/dl | Pre<br>1 w<br>5 w | | | 190.64 ± 4.63 (n = 14)<br>190.43 ± 6.82 (n = 14)<br>182.93 ± 5.89 (n = 14)<br>Friedman test: NS | Friedman test |
| Fig. S2<br>(Upper right, DM) | mg/dl | Pre<br>DM 1w<br>DM 5w | | | 193.64 ± 5.06 (n = 14)<br>527.93 ± 18.80 (n = 14)<br>519.14 ± 18.92 (n = 14)<br>Friedman test: *** | Friedman test<br>post-hoc Conover's multiple comparison test with bonferroni's correction |
| Fig. S2<br>(Bottom left, DM + S) | mg/dl | Pre<br>DM 1w<br>DM 5w + S 1d<br>DM 5w + S 2d<br>DM 5w + S 3d | | | 203.33 ± 4.66 (n = 9)<br>545.44 ± 18.00 (n = 9)<br>595.67 ± 3.54 (n = 9)<br>586.56 ± 4.09 (n = 9)<br>598.89 ± 1.11 (n = 9)<br>Friedman test: *** | Friedman test<br>post-hoc Conover's multiple comparison test with bonferroni's correction |
| Fig. S2<br>(Bottom right, DM + L) | mg/dl | Pre<br>DM 1w<br>DM 5w + L 1d<br>DM 5w + L 2d<br>DM 5w + L 3d | | | 192.20 ± 6.07 (n = 10)<br>578.50 ± 12.92 (n = 10)<br>586.50 ± 8.33 (n = 10)<br>555.00 ± 15.13 (n = 10)<br>566.80 ± 13.40 (n = 10)<br>Friedman test: *** | Friedman test<br>post-hoc Conover's multiple comparison test with bonferroni's correction |
| Fig. S3C | % | CTL_Sup<br>DM_Sup<br>CTL_Med<br>DM_Med<br>CTL_Dep<br>DM_Dep | | 6.96 ± 0.66 (n = 16)<br>9.88 ± 1.16 (n = 16)<br>1.37 ± 0.24 (n = 16)<br>1.32 ± 0.29 (n = 16)<br>0.32 ± 0.15 (n = 16)<br>0.098 ± 0.067 (n = 16)<br>Kruskal-Wallis test: *** | 6.96 ± 0.87 (n = 4)<br>9.88 ± 1.93 (n = 4)<br>1.37 ± 0.35 (n = 4)<br>1.32 ± 0.38 (n = 4)<br>0.32 ± 0.19 (n = 4)<br>0.098 ± 0.098 (n = 4)<br>Kruskal-Wallis test: *** | Kruskal-Wallis test<br>post-hoc Dunn's multiple comparison test with bonferroni's correction |
| Fig. S3D | µm | CTL_CD169-<br>CTL_CD169+<br>DM_CD169-<br>DM_CD169+ | 67.84 ± 3.34 (n = 15)<br>28.49 ± 2.24 (n = 15)<br>64.39 ± 4.18 (n = 15)<br>28.20 ± 1.86 (n = 15)<br>Kruskal-Wallis test: *** | | 67.62 ± 1.15 (n = 4)<br>28.58 ± 0.55 (n = 4)<br>63.51 ± 4.53 (n = 4)<br>28.20 ± 0.94 (n = 4)<br>Kruskal-Wallis test: ** | Kruskal-Wallis test<br>post-hoc Dunn's multiple comparison test with bonferroni's correction |

*Continued*

| Figure | Unit | Group | Mean ± SEM<br>(per cell) | Mean ± SEM<br>(per FOV) | Mean ± SEM<br>(per animal) | TEST |
|---|---|---|---|---|---|---|
| Fig. S4A<br>(Cell density) | cells/FOV | CTL<br>DM | | 16.08 ± 1.18 (n = 12)<br>17.56 ± 1.06 (n = 9)<br>NS | 15.22 ± 1.49 (n = 6)<br>17.10 ± 1.21 (n = 5)<br>NS | Welch's T test |
| Fig. S4A<br>(Cell body size) | μm$^2$ | CTL<br>DM | 38.96 ± 1.36 (n = 35)<br>47.05 ± 1.95 (n = 30)<br>** | | 38.52 ± 1.76 (n = 6)<br>46.06 ± 2.06 (n = 5)<br>* | Welch's T test |
| Fig. S4A<br>(Territory area) | μm$^2$ | CTL<br>DM | 3727.18 ± 187.54 (n = 35)<br>3395.37 ± 287.96 (n = 30)<br>NS | | 3776.20 ± 296.48 (n = 6)<br>3221.46 ± 437.94 (n = 5)<br>NS | Welch's T test |
| Fig. S4A<br>(Primary process length) | μm | CTL<br>DM | 57.22 ± 2.05 (n = 35)<br>55.63 ± 2.20 (n = 30)<br>NS | | 55.85 ± 2.49 (n = 6)<br>55.70 ± 2.98 (n = 5)<br>NS | Welch's T test |
| Fig. S4A<br>(Total process length) | μm | CTL<br>DM | 366.83 ± 11.90 (n = 35)<br>357.12 ± 15.05 (n = 30)<br>NS | | 371.24 ± 12.10 (n = 6)<br>349.96 ± 15.29 (n = 5)<br>NS | Welch's T test |
| Fig. S4B<br>(Cell density) | cells/FOV | CTL<br>DM | | 16.27 ± 1.30 (n = 11)<br>18.54 ± 0.92 (n = 11)<br>NS | 16.47 ± 1.37 (n = 6)<br>18.69 ± 0.96 (n = 8)<br>NS | Welch's T test |
| Fig. S4B<br>(Cell body size) | μm$^2$ | CTL<br>DM | 41.33 ± 1.49 (n = 37)<br>45.34 ± 1.45 (n = 42)<br>NS | | 41.00 ± 2.02 (n = 6)<br>46.15 ± 1.64 (n = 8)<br>NS | Welch's T test |
| Fig. S4B<br>(Territory area) | μm$^2$ | CTL<br>DM | 3904.85 ± 272.08 (n = 37)<br>2919.15 ± 172.41 (n = 42)<br>** | | 3869.27 ± 408.89 (n = 6)<br>2838.19 ± 270.48 (n = 8)<br>NS | Welch's T test |
| Fig. S4B<br>(Primary process length) | μm | CTL<br>DM | 57.07 ± 1.88 (n = 37)<br>49.01 ± 1.62 (n = 42)<br>** | | 56.54 ± 2.11 (n = 6)<br>50.44 ± 2.28 (n = 8)<br>NS | Welch's T test |
| Fig. S4B<br>(Total process length) | μm | CTL<br>DM | 353.23 ± 16.02 (n = 37)<br>304.38 ± 12.40 (n = 42)<br>* | | 351.66 ± 26.79 (n = 6)<br>313.78 ± 13.91 (n = 8)<br>NS | Welch's T test |
| Fig. S4C<br>(Cell density) | cells/FOV | CTL<br>DM | | 4.70 ± 0.37 (n = 20)<br>4.55 ± 0.41 (n = 20)<br>NS | 4.70 ± 0.41 (n = 5)<br>4.65 ± 0.26 (n = 5)<br>NS | Mann Whitney U test |
| Fig. S4C<br>(Cell body size) | μm$^2$ | CTL<br>DM | 40.25 ± 0.94 (n = 85)<br>42.56 ± 1.23 (n = 88)<br>NS | | 39.75 ± 1.82 (n = 5)<br>42.90 ± 1.47 (n = 5)<br>NS | Welch's T test |
| Fig. S4C<br>(Territory area) | μm$^2$ | CTL<br>DM | 6499.19 ± 358.63 (n = 66)<br>5988.49 ± 351.63 (n = 68)<br>NS | | 6479.43 ± 271.04 (n = 5)<br>6009.56 ± 393.52 (n = 5)<br>NS | Welch's T test |
| Fig. S4C<br>(Primary process length) | μm | CTL<br>DM | 71.54 ± 2.56 (n = 66)<br>67.72 ± 2.37 (n = 68)<br>NS | | 71.50 ± 2.04 (n = 5)<br>67.75 ± 3.08 (n = 5)<br>NS | Welch's T test |
| Fig. S4C<br>(Total process length) | μm | CTL<br>DM | 552.40 ± 23.61 (n = 66)<br>514.23 ± 24.50 (n = 68)<br>NS | | 549.90 ± 25.61 (n = 5)<br>517.21 ± 36.92 (n = 5)<br>NS | Welch's T test |
| Fig. S4D<br>(Cell density) | cells/FOV | CTL<br>DM | | 5.75 ± 0.37 (n = 20)<br>7.00 ± 0.48 (n = 20)<br>NS | 5.75 ± 0.40 (n = 5)<br>7.00 ± 0.45 (n = 5)<br>NS | Mann Whitney U test |
| Fig. S4D<br>(Cell body size) | μm$^2$ | CTL<br>DM | 39.86 ± 0.80 (n = 110)<br>42.25 ± 0.90 (n = 128)<br>* | | 40.07 ± 2.07 (n = 5)<br>42.29 ± 2.31 (n = 5)<br>NS | Welch's T test |
| Fig. S4D<br>(Territory area) | μm$^2$ | CTL<br>DM | 6724.23 ± 245.52 (n = 76)<br>5886.57 ± 286.91 (n = 85)<br>* | | 6648.21 ± 436.91 (n = 5)<br>5786.12 ± 384.32 (n = 5)<br>NS | Welch's T test |
| Fig. S4D<br>(Primary process length) | μm | CTL<br>DM | 70.53 ± 1.92 (n = 76)<br>61.54 ± 1.92 (n = 85)<br>** | | 70.14 ± 2.23 (n = 5)<br>61.03 ± 1.66 (n = 5)<br>* | Welch's T test |
| Fig. S4D<br>(Total process length) | μm | CTL<br>DM | 644.91 ± 49.68 (n = 76)<br>557.84 ± 22.01 (n = 85)<br>** | | 637.44 ± 41.80 (n = 5)<br>545.25 ± 43.99 (n = 5)<br>NS | Welch's T test |
| Fig. S4E<br>(Cell density) | cells/FOV | CTL<br>DM | | 8.85 ± 0.39 (n = 20)<br>9.45 ± 0.33 (n = 20)<br>NS | 8.85 ± 0.23 (n = 5)<br>9.45 ± 0.41 (n = 5)<br>NS | Mann Whitney U test |
| Fig. S4E<br>(Cell body size) | μm$^2$ | CTL<br>DM | 36.62 ± 0.65 (n = 155)<br>40.10 ± 0.66 (n = 165)<br>*** | | 36.06 ± 1.06 (n = 5)<br>39.94 ± 0.96 (n = 5)<br>NS | Welch's T test |
| Fig. S4E<br>(Territory area) | μm$^2$ | CTL<br>DM | 6209.12 ± 223.87 (n = 98)<br>5095.78 ± 177.72 (n = 114)<br>*** | | 6180.44 ± 130.09 (n = 5)<br>4939.20 ± 541.67 (n = 5)<br>NS | Welch's T test |
| Fig. S4E<br>(Primary process length) | μm | CTL<br>DM | 65.23 ± 1.51 (n = 98)<br>55.27 ± 1.26 (n = 114)<br>*** | | 64.54 ± 1.52 (n = 5)<br>54.74 ± 2.32 (n = 5)<br>* | Welch's T test |
| Fig. S4E<br>(Total process length) | μm | CTL<br>DM | 598.87 ± 15.81 (n = 98)<br>517.95 ± 15.07 (n = 114)<br>*** | | 591.99 ± 24.30 (n = 5)<br>497.84 ± 54.95 (n = 5)<br>NS | Welch's T test |

*Continued*

| Figure | Unit | Group | Mean ± SEM (per cell) | Mean ± SEM (per FOV) | Mean ± SEM (per animal) | TEST |
|---|---|---|---|---|---|---|
| Fig. S5C | μm$^2$/cell | WT<br>Cx3cr1$^{GFP/+}$ | 10.66 ± 0.52 (n = 111)<br>9.71 ± 0.50 (n = 130)<br>NS | | 10.78 ± 0.66 (n = 5)<br>9.64 ± 0.86 (n = 5)<br>NS | Welch's T test |
| Fig. S5D | μm$^2$/cell | CTL<br>DM<br>DM+L<br>LPS | 9.71 ± 0.50 (n = 130)<br>14.55 ± 0.72 (n = 143)<br>11.50 ± 0.58 (n = 150)<br>16.06 ± 1.07 (n = 173)<br>One-way ANOVA: *** | | 9.64 ± 0.86 (n = 5)<br>14.5 ± 1.34 (n = 5)<br>11.66 ± 1.28 (n = 5)<br>16.09 ± 2.53 (n = 5)<br>One-way ANOVA: NS | One-way ANOVA Bonferroni's post hoc test |
| Fig. S6 | μm | CTL_Extension<br>DM_Extension<br>CTL_Retraction<br>DM_Retraction | 7.92 ± 0.33 (n = 58)<br>8.02 ± 0.37 (n = 49)<br>7.93 ± 0.37 (n = 58)<br>9.38 ± 0.37 (n =49)<br>Two-way ANOVA:<br>Extension vs Retraction: NS<br>CTL vs DM: *<br>interaction (Direction x group): NS | | 7.83 ± 0.65 (n = 7)<br>7.83 ± 0.50 (n = 7)<br>7.88 ± 0.70 (n = 7)<br>9.26 ± 0.46 (n = 7)<br>Two-way ANOVA:<br>Extension vs Retraction: NS<br>CTL vs DM: NS<br>interaction (Direction x group): NS | Two-way ANOVA Bonferroni's post hoc test |
| Fig. S7A | cells/FOV | CTL<br>LPS | | 16.17 ± 0.86 (n = 23)<br>18.58 ± 0.85 (n = 12)<br>NS | 16.48 ± 1.08 (n = 9)<br>18.80 ± 0.82 (n = 5)<br>NS | Welch's T test |
| Fig. S7B | μm$^2$ | CTL<br>LPS | 40.18 ± 1.01 (n = 72)<br>46.27 ± 1.54 (n = 33)<br>** | | 39.08 ± 1.56 (n = 9)<br>46.65 ± 2.06 (n = 5)<br>* | Welch's T test |
| Fig. S7C | μm$^2$ | CTL<br>LPS | 3818.49 ± 166.10 (n = 72)<br>2975.13 ± 143.35 (n = 33)<br>*** | | 3772.73 ± 246.02 (n = 9)<br>2998.12 ± 227.40 (n = 5)<br>* | Welch's T test |
| Fig. S7D | μm | CTL<br>LPS | 57.14 ± 1.38 (n = 72)<br>44.13 ± 1.79 (n = 33)<br>*** | | 56.43 ± 1.69 (n = 9)<br>44.83 ± 1.83 (n = 5)<br>*** | Welch's T test |
| Fig. S7E | μm | CTL<br>LPS | 359.84 ± 10.02 (n = 72)<br>274.95 ± 13.27 (n = 33)<br>*** | | 366.62 ± 16.64 (n = 9)<br>270.56 ± 12.65 (n = 5)<br>*** | Welch's T test |
| Fig. S7F | μm$^2$ | CTL<br>LPS | 52.06 ± 2.47 (n = 58)<br>61.45 ± 3.94 (n = 32)<br>* | | 51.55 ± 2.74 (n = 7)<br>60.76 ± 4.63 (n = 5)<br>NS | Welch's T test |
| Fig. S7G | μm | CTL<br>LPS | 1225.38 ± 92.01 (n = 58)<br>1081.80 ± 85.14 (n = 32)<br>NS | | 1213.26 ± 185.27 (n = 7)<br>1080.77 ± 90.21 (n = 5)<br>NS | Welch's T test |
| Fig. S7H | μm | CTL<br>LPS | 15.96 ± 0.64 (n = 58)<br>19.28 ± 0.72 (n = 32)<br>*** | | 15.81 ± 1.25 (n = 7)<br>19.37 ± 0.86 (n = 5)<br>NS | Welch's T test |
| Fig. S7I | μm$^2$ | CTL<br>LPS | 12.27 ± 1.14 (n = 58)<br>17.42 ± 1.36 (n = 32)<br>** | | 11.99 ± 2.15 (n = 7)<br>17.54 ± 2.11 (n = 5)<br>NS | Welch's T test |
| Fig. S7J | cells/FOV | CTL<br>LPS | | 10.45 ± 0.56 (n= 20)<br>11.10 ± 0.56 (n = 20)<br>NS | 10.45 ± 0.58 (n = 5)<br>11.10 ± 0.28 (n = 5)<br>NS | Welch's T test |
| Fig. S7K | μm$^2$ | CTL<br>LPS | 40.03 ± 0.61 (n = 195)<br>47.93 ± 2.54 (n = 101)<br>** | | 39.92 ± 1.82 (n = 5)<br>47.24 ± 7.73 (n = 5)<br>NS | Welch's T test |
| Fig. S7L | μm$^2$ | CTL<br>LPS | 6619.63 ± 211.69 (n = 142)<br>4585.65 ± 227.04 (n = 114)<br>*** | | 6585.54 ± 296.44 (n = 5)<br>4582.26 ± 618.68 (n = 5)<br>* | Welch's T test |
| Fig. S7M | μm | CTL<br>LPS | 71.00 ± 1.57 (n = 142)<br>46.83 ± 1.70 (n = 114)<br>*** | | 70.91 ± 1.72 (n = 5)<br>46.78 ± 2.56 (n = 5)<br>*** | Welch's T test |
| Fig. S7N | μm | CTL<br>LPS | 601.91 ± 15.65 (n = 142)<br>478.89 ± 17.83 (n = 114)<br>*** | | 597.61 ± 30.33 (n = 5)<br>478.27 ± 43.07 (n = 5)<br>NS | Welch's T test |
| Fig. S8A | cells/FOV | DM + S<br>DM + L | | 12.35 ± 0.43 (n = 20)<br>9.70 ± 0.36 (n = 20)<br>*** | 12.35 ± 0.44 (n = 5)<br>9.70 ± 0.41 (n = 5)<br>** | Mann Whitney U test |
| Fig. S8B | μm$^2$ | DM + S<br>DM + L | 45.27 ± 1.70 (n = 104)<br>44.77 ± 1.52 (n = 91)<br>NS | | 45.08 ± 3.44 (n = 5)<br>45.25 ± 3.28 (n = 5)<br>NS | Welch's T test |
| Fig. S8C | μm$^2$ | DM + S<br>DM + L | 6023.78 ± 252.07 (n = 111)<br>8054.93 ± 388.29 (n = 91)<br>*** | | 6045.31 ± 300.98 (n = 5)<br>8152.41 ± 1273.33 (n = 5)<br>NS | Welch's T test |
| Fig. S8D | μm | DM + S<br>DM + L | 57.48 ± 1.56 (n = 111)<br>72.13 ± 2.22 (n = 91)<br>*** | | 57.51 ± 1.68 (n = 5)<br>72.86 ± 3.87 (n = 5)<br>* | Welch's T test |
| Fig. S8E | μm | DM + S<br>DM + L | 551.63 ± 18.25 (n = 111)<br>758.96 ± 23.85 (n = 91)<br>*** | | 553.88 ± 21.23 (n = 5)<br>768.25 ± 60.97 (n = 5)<br>* | Welch's T test |

| *Continued* | | | | | | |
|---|---|---|---|---|---|---|
| Figure | Unit | Group | Mean ± SEM (per cell) | Mean ± SEM (per FOV) | Mean ± SEM (per animal) | TEST |
| Fig. S9A | $\mu m^2$ | CTL<br>Liraglutide | 52.06 ± 2.47 (n = 58)<br>54.05 ± 1.79 (n = 39)<br>NS | | 51.55 ± 2.74 (n = 7)<br>54.12 ± 1.17 (n = 5)<br>NS | Welch's T test |
| Fig. S9B | $\mu m^2$ | CTL<br>Liraglutide | 1225.38 ± 92.01 (n = 58)<br>1214.17 ± 104.03 (n = 39)<br>NS | | 1213.26 ± 185.27 (n = 7)<br>1223.05 ± 106.36 (n = 5)<br>NS | Welch's T test |
| Fig. S9C | µm | CTL<br>Liraglutide | 15.96 ± 0.64 (n = 58)<br>14.08 ± 0.44 (n = 39)<br>* | | 15.81 ± 1.25 (n = 7)<br>14.17 ± 0.98 (n = 5)<br>NS | Welch's T test |
| Fig. S9D | $\mu m^2$ | CTL<br>Liraglutide | 12.27 ± 1.14 (n = 58)<br>9.16 ± 1.02 (n = 39)<br>* | | 11.99 ± 2.15 (n = 7)<br>9.26 ± 1.40 (n = 5)<br>NS | Welch's T test |
| Fig. S9E | cells/FOV | CTL<br>Liraglutide | | 10.45 ± 0.56 (n= 20)<br>12.83 ± 1.29 (n = 12)<br>NS | 10.45 ± 0.58 (n = 5)<br>12.83 ± 2.71 (n = 3)<br>NS | Welch's T test |
| Fig. S9F | $\mu m^2$ | CTL<br>Liraglutide | 40.03 ± 0.61 (n = 195)<br>39.38 ± 1.32 (n = 52)<br>NS | | 39.92 ± 1.82 (n = 5)<br>39.71 ± 1.84 (n = 3)<br>NS | Welch's T test |
| Fig. S9G | $\mu m^2$ | CTL<br>Liraglutide | 6619.63 ± 211.69 (n = 142)<br>6890.16 ± 406.89 (n = 54)<br>NS | | 6585.54 ± 296.44 (n = 5)<br>7133.87 ± 1186.93 (n = 3)<br>NS | Welch's T test |
| Fig. S9H | $\mu m^2$ | CTL<br>Liraglutide | 71.00 ± 1.57 (n = 142)<br>58.40 ± 2.66 (n = 54)<br>*** | | 70.91 ± 1.72 (n = 5)<br>59.21 ± 5.22 (n = 3)<br>NS | Welch's T test |
| Fig. S9I | $\mu m^2$ | CTL<br>Liraglutide | 601.91 ± 15.65 (n = 142)<br>621.14 ± 26.81 (n = 54)<br>NS | | 597.61 ± 30.33 (n = 5)<br>636.35 ± 69.06 (n = 3)<br>NS | Welch's T test |
| Fig. S10B | cells/FOV | CTL<br>DM<br>DM+L | | 15.75 ± 1.08 (n = 8)<br>15.63 ± 0.82 (n = 8)<br>16.75 ± 0.80 (n = 8)<br>One-way ANOVA: NS | 15.75 ± 0.75 (n = 2)<br>15.63 ± 0.88 (n = 2)<br>16.75 ± 0.50 (n = 2)<br>One-way ANOVA: NS | One-way ANOVA |

**Table S3.** Quantitative data corresponding to the analyses presented in Figs. 1-4 and Figs. S1-S10. For each figure, the number of animals, the number of fields of view (FOVs), and the number of microglial cells analyzed are also indicated. Data are presented as mean ± SEM.

**Movie S1.** Representative movie from two-photon microscopy observations of retinal microglia (labeled with GFP) and blood vessels (labeled with Evans blue) in a Cx3cr1$^{GFP/+}$ mouse (control). The movie captures 30 minutes of continuous imaging, demonstrating the dynamic behavior of microglial processes. The inset highlights an enlarged view of the movements of a single microglial process within the area indicated by a white rectangle.

**Movie S2.** Representative movies showing the dynamic movements of retinal microglial processes in control (CTL) and diabetic (DM) mice. The movies capture 10 minutes of continuous retinal imaging. Green lines indicate the trajectories connecting the initial (yellow dots) and end points (red dots) of individual microglial process tips. Yellow polygons indicate the surveillance territories of individual microglial process tips during the imaging period.

**Validation of two-photon microscopy data (conversion from pixel to micrometer units)**

In this study, we used a commercially available glycerin immersion objective lens with a custom-made contact lens. The refractive indices of internal ocular components, such as the crystalline lens and vitreous body, as well as the optical properties of these components, could potentially affect the scaling of the two-photon microscopy images. To convert pixel units of two-photon microscopy images into micrometer (µm) units, we utilized the cell body size as a reference among microglial morphological parameters, which include cell body size, territory area, primary process length, and total process length, as shown in Fig. 2 *B-F*. The cell body size was less affected by imaging modality compared with microglial process lengths, which tended to appear shorter in two-photon images than in confocal images. This difference was likely due to the nature of imaging techniques: two-photon images were obtained from the fixed Z-plane sections, whereas confocal images were reconstructed from Z-stacked images.

The conversion from pixel to micrometer units was evaluated through the following steps:

Step 1: Assessment of paraformaldehyde-induced retinal shrinkage

We first assessed the potential effect of paraformaldehyde (PFA) solution on retinal shrinkage in flat-mounted preparations. The anteroposterior diameter of the mouse eyeball was 3.36 ± 0.05 mm without PFA perfusion, and 3.39 ± 0.04 mm 24 hours after PFA perfusion (mean ± SEM, five samples from different mice in each group). These results suggest that PFA-induced shrinkage does not significantly affect the measurements of microglial morphological parameters.

Step 2: Calculation of conversion coefficient

We then calculated the square root of the mean cell body size (in pixels$^2$) from two-photon microscopy images, using samples from three cohorts: Fig. 2*C* (CTL and DM groups) and Fig. 7*B* (LPS group). The values were 16.68 pixels for the CTL group, 17.86 pixels for the DM group, and 17.90 pixels for the LPS group. Next, we calculated the square root of the mean cell body size (in µm$^2$) of confocal microscopy images in the corresponding cohorts: Fig. 2*J* (CTL and DM groups) and Fig. 7*K* (LPS group). The values were 6.33 µm for the CTL group, 6.51 µm for the DM group, and 6.92 µm for the LPS group.

Step 3: Determination of the conversion factor

We divided the square root values obtained from confocal data by those from two-photon data to determine the conversion factors: 0.38 µm/pixel for the CTL group, 0.36 µm/pixel for the DM group, and 0.39 µm/pixel for the LPS group. Finally, we averaged these values to determine the final conversion coefficient, which was calculated to be 0.38 µm/pixel. This coefficient was uniformly applied for converting pixel-based measurements obtained from two-photon microscopy into µm units, ensuring consistency with the spatial resolution observed in confocal microscopy.
